# Interplay of Interlocus Gene Conversion and Crossover in Segmental Duplications Under a Neutral Scenario

**DOI:** 10.1534/g3.114.012435

**Published:** 2014-06-06

**Authors:** Diego A. Hartasánchez, Oriol Vallès-Codina, Marina Brasó-Vives, Arcadi Navarro

**Affiliations:** * Institute of Evolutionary Biology (Universitat Pompeu Fabra – CSIC), PRBB, Barcelona, Catalonia, Spain, 08003; † National Institute for Bioinformatics (INB), Barcelona, Catalonia, Spain, 08003; ‡ Institució Catalana de Recerca i Estudis Avançats (ICREA), Barcelona, Catalonia, Spain, 08010; § Centre for Genomic Regulation (CRG), Barcelona, Catalonia, Spain, 08003

**Keywords:** concerted evolution, linkage disequilibrium, recombination hotspots, forward simulations, increased variation

## Abstract

Interlocus gene conversion is a major evolutionary force that drives the concerted evolution of duplicated genomic regions. Theoretical models successfully have addressed the effects of interlocus gene conversion and the importance of crossover in the evolutionary fate of gene families and duplications but have not considered complex recombination scenarios, such as the presence of hotspots. To study the interplay between interlocus gene conversion and crossover, we have developed a forward-time simulator that allows the exploration of a wide range of interlocus gene conversion rates under different crossover models. Using it, we have analyzed patterns of nucleotide variation and linkage disequilibrium within and between duplicate regions, focusing on a neutral scenario with constant population size and validating our results with the existing theoretical models. We show that the interaction of gene conversion and crossover is nontrivial and that the location of crossover junctions is a fundamental determinant of levels of variation and linkage disequilibrium in duplicated regions. We also show that if crossover activity between duplications is strong enough, recurrent interlocus gene conversion events can break linkage disequilibrium within duplicates. Given the complex nature of interlocus gene conversion and crossover, we provide a framework to explore their interplay to help increase knowledge on molecular evolution within segmental duplications under more complex scenarios, such as demographic changes or natural selection.

Gene duplication has been recognized as a primary source of genetic innovation since Ohno’s seminal work on this topic in 1970 (Ohno 1970). The accumulation of genomic sequence data from a wide range of species has shown that segmental duplications (SDs) spanning more than 1 kb and retaining a high degree of sequence homogeneity (>90%) are a pervasive feature of eukaryotic genomes (Lynch and Conery 2000; Bailey *et al.* 2002; Marques-Bonet *et al.* 2009). Understanding the molecular evolution of duplicated nucleotide sequences is also of great relevance because SDs define hotspots of chromosomal rearrangements and are known to give rise to copy-number variants (Sharp *et al.* 2005; Mills *et al.* 2011; Uddin *et al.* 2011), which in turn frequently are implicated in disease susceptibility (Conrad *et al.* 2010; Stankiewicz and Lupski 2010) and are targets of natural selection (Gazave *et al.* 2011; Iskow *et al.* 2012; Lorente-Galdos *et al.* 2013).

During the 1980s, extensive theoretical work was carried out on the subject of multigene family evolution (Baltimore 1981; Dover 1982; Ohta 1982, 1983; Nagylaki and Petes 1982; Nagylaki 1984). More recently, Innan and collaborators (Innan 2002, 2003; Teshima and Innan 2004; Mano and Innan 2008) and Thornton (2007) have successfully combined analytical results and coalescent simulations to study diversity patterns and divergence times between duplicates and have analyzed various selection scenarios. Still, many unique features of SDs have not yet been fully addressed, neither analytically or by simulation.

The foremost feature of duplicated genomic regions is that they undergo concerted evolution due to the exchange of genetic information via interlocus gene conversion (IGC) (Nagylaki and Petes 1982; Ohta 1982). IGC can be described as a copy-paste event (Innan 2009) whereby a fragment of one of the duplicated segments is copied onto the corresponding segment of the duplicate (Wiuf and Hein 2000). IGC is also referred to as nonallelic, interparalog, or ectopic gene conversion. IGC is known to be a major player in small (two-copy) multigene family evolution (Ohta 1983), and empirical estimates of IGC rates span several orders of magnitude (Chen *et al.* 2007; Benovoy and Drouin 2009; McGrath *et al.* 2009). The extent to which the theory from multigene family evolution can be applied to the evolution of SDs and copy-number variants remains an open question. In particular, because SDs can span large regions, some genomic features, such as the pervasiveness of crossover hotspots in the human genome (Jeffreys *et al.* 2001; Kong *et al.* 2002), might have non-negligible effects on the levels of variation within and between SDs. The evolution of SDs under different crossover models, including the overlap of IGC susceptible regions with crossover hotspots, or the linkage disequilibrium (LD) patterns within and between SDs, are some of the issues that, to our knowledge, have not been addressed before.

To tackle some of these questions, a flexible forward-time simulator should prove to be a powerful tool. Although coalescent simulators have the advantage of being extremely fast, forward-time simulators allow the exploration of a wider parameter space and the implementation of different scenarios (such as sophisticated crossover models) in a straightforward way. We here introduce a forward-time algorithm in C++ and explore a wide range of realistic parameter values in a model of neutral evolution of duplicated chromosomal regions undergoing mutation, IGC, and crossover under different models.

## Materials and Methods

We model a Wright-Fisher population of *N* diploid individuals, where each of the *2N* chromosomes consists of either two or three blocks. Each block is *L* nucleotides long and is characterized by an ordered set of integers (representing nucleotide positions) between 0 and *L−1*.

### Simulation procedure: phases

**Figure 1:**
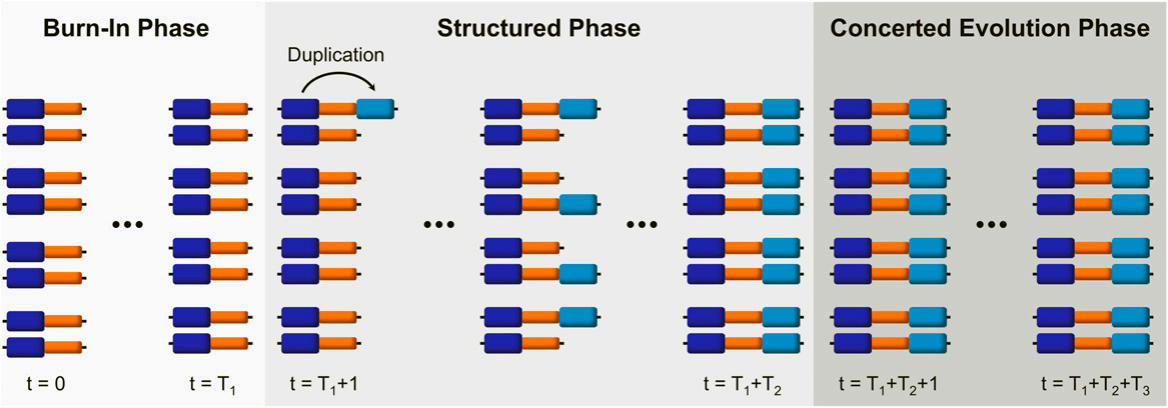
The three phases of every simulation run. Each simulation begins with a burn-in phase, in which a population formed by chromosomes with two single-copy blocks (dark blue and orange) is brought to mutation-drift equilibrium. The duplication of the first of these blocks (original block: dark blue, duplicated block: light blue) marks the initiation of the structured phase, during which the duplication becomes fixed. Finally, during the concerted evolution phase, the population reaches a new equilibrium in which the interplay of interlocus gene conversion between duplicated blocks and crossover determines levels and patterns of variability.

Simulations have three phases, the *burn-in phase*, the *structured phase*, and the *concerted evolution phase*. Each simulation begins with a burn-in phase of *T_1_* generations in which the population undergoes random mating, mutation, and recombination by crossover. During this first phase, each chromosome is composed of two blocks that we will refer to as *original* and *single-copy* blocks. We run this first phase long enough for the population to reach equilibrium. At *t = T_1_* + 1, the structured phase starts with a duplication event in which the original block of a randomly chosen chromosome is duplicated and “pasted” to the right of its single-copy block (Figure 1). We shall refer to this third block as *duplicated* block. This unique duplication is fixed by drift. To achieve fixation in a computationally efficient way, a neutral fixation trajectory is simulated using Kimura’s pseudo-sampling method (Kimura 1980). During the structured phase, of average *T_2_* = 4*N* generations (and maximum *T_2_* = 20*N* generations), the number of chromosomes carrying the duplication in each generation is determined by the fixation trajectory. Once the fixation of the duplication has occurred, the program proceeds to a concerted evolution phase (of *T_3_* = *T_T_* – *T_1_* – *T_2_* generations) until *t = T_T_*.

### Mutation

Mutation is active during all phases and occurs independently in each block at a rate μ per site per generation. We use a pseudo infinite-sites model (Padhukasahasram *et al.* 2008) in which the number of sites in each block is finite (*L*), but mutations can only appear in nonpolymorphic positions. For mutational purposes, a variant that occurs in either the original or the duplicated block is still considered as polymorphic even if it is segregating only in one of them, and therefore any mutation is only considered as *fixed* if it is present in all chromosomes on both blocks. Multiple mutations are not allowed in the same block and generation. We have verified that allowing multiple mutations does not change the results presented in this work.

### Crossover

Homologous crossover occurs at rate *r* per meiosis, with a maximum of one crossover allowed per meiosis. Most previous studies of multigene family evolution (Ohta 1983; Nagylaki 1984; Innan 2002; Thornton 2007) consider intergenic crossover only. In other words, previous models are such that meiotic crossover junctions are only allowed between duplicated blocks (usually called “genes” in these papers) but not within them. We will refer to this model as the single-copy crossover (SCC) model since all crossover junctions fall within the single-copy block. Here, we explore two additional crossover models: the whole-region crossover (WRC) model, that allows crossover junctions from 5′ end of one duplicate to 3′ end of the other duplicate, which so far has only been used by Teshima and Innan (2012); and the hotspot crossover (HSC) model, in which a particular region of the simulated segment is predefined as a crossover hotspot such that all crossover junctions fall within this region. Choosing an adequate definition of *r* is not straightforward, given that chromosomes with different lengths (two or three blocks) segregate at changing frequencies in the population, and that different models have different *a priori* distributions of crossover events. To facilitate comparison among models, we define *r* per meiosis, irrespective of the lengths of the chromosomes involved. In other words, the same average number of crossovers occurs per meiosis per generation under any model and phase. This implies, for example, that under the WRC model, the effective crossover rate per base pair is higher during the burn-in phase (two blocks) than during the concerted evolution phase (three blocks). The effects of this choice are always small, do not affect measures taken at equilibrium, and are easier to account for than those that would be introduced by other potential definitions of *r*. We will refer to the population scaled crossover rate as *R* = 4N*r*. At each crossover event, a crossover junction is randomly selected from the region allowed by the underlying crossover model.

### Interlocus gene conversion

At every generation, if a chromosome contains the duplication, there is a per site probability of an IGC event being initiated, *g*. An IGC event can be described as a copy-paste event (Innan 2009) in which the information (*i.e.*, all derived mutations) contained along an IGC tract on one of the duplicate blocks is copied and pasted onto the other duplicate substituting any variants that were previously within the paralogous tract in that duplicate. In our model, an IGC event can be initiated at any position of any duplicate block according to a uniform distribution. The length of IGC tracts is determined according to a geometric distribution with average size *λ*, which fits available empirical evidence (Wiuf and Hein 2000). Following Wiuf and Hein (2000), the tract length for every IGC event, *l*, is extracted from a geometric distribution with parameter *q = λ/L:*

$$P_{q}\left(l\right)=q{\left(1-q\right)}^{l-1}.$$ (1)

Instead of randomly choosing the direction in which the IGC tract extends (either 3′ or 5′), we determine the IGC tract extending *l/2* sites to the left and *l/2* to the right of the initiation point or junction. Mansai *et al.* (2011) proposed a similar model in which there is an independent exponential elongation of the gene conversion tract in both directions from the initiation point. We deviate from previous models (Wiuf and Hein 2000; Thornton 2007; Mansai *et al.* 2011) in limiting IGC tracts to the duplicate regions by the simple procedure of truncating any tract that extends beyond the duplicate blocks. We expect that this simplification has a negligible impact on our results. In our model, IGC only occurs between paralogous regions on the same chromosome and both copies act as donor or receptor with equal probability. The mutation positions found in the donor tract are imported to the receptor, and the mutations in the receptor tract are erased. Finally, we allow IGC to occur regardless of the divergence between blocks on the same chromosome (Supporting Information, File S1). Other authors, notably Teshima and Innan (2004), implemented a divergence threshold above which IGC is terminated. The IGC rate per site per generation will be *c* = *gλ*, where *λ* is the effective average length of the IGC tracts (although not accounting for possible truncations) and the population IGC rate will be *C =* 4*Nc*.

### Eras: simulating neutral genealogies

The neutral scenario allows us to decrease the running time of simulations by avoiding the explicit simulation of chromosomes that will eventually leave no offspring in the population (Padhukasahasram *et al.* 2008). This requires simulating a genealogy for *k* generations in advance and then tracing it back to ascertain which chromosomes, at each generation, will make a contribution to the final population. For the sake of clarity, we will refer to each period of *k* generations as an *Era*. Because there is a trade-off between the time it takes to simulate the genealogy and the time saved by not simulating those chromosomes destined to be lost, we need to choose an optimal value of *k*. This value varies considerably with population size, crossover rate, and other parameters, so we selected *k* by performing trial runs for every combination of parameters (not shown).

Eras start at generation *t’ =* 0. To construct the genealogy for each Era, each chromosome at each of the *k* generations is randomly assigned a *parental* chromosome from the previous generation. When *R* = 0, the process is straightforward: first, all chromosomes at generation *t’ = k* − 1, are tagged as *fertile*; next, the parental chromosome of every fertile individual at *t’ = k* − 1 is tagged as fertile at *t’ = k* − 2; and the same is repeated for every generation until *t*’ = 0. The case with crossover (*R >* 0) is essentially the same except that when tagging the parental chromosomes as fertile, there is a probability, *r*, that the *partner* of that chromosome (*i.e.*, the other chromosome from the same individual) is also tagged as fertile.

During the structured phase, the number of chromosomes carrying the duplication (*s(t’))* is determined by a neutral fixation trajectory. As mentioned previously, the structured phase begins with a unique duplication event in a randomly chosen chromosome (*i.e. s*(*t’=0*) = 1). At *t’ = 1*, *s(1)* randomly chosen chromosomes will be assigned the chromosome carrying the duplication as their parental chromosome. The rest of the 2*N – s(1)* chromosomes will be randomly assigned a parental chromosome not carrying the duplication (at this point, any other chromosome). At *t*’ = 2, *s*(2) randomly chosen chromosomes will be assigned any of the *s(1)* chromosomes carrying the duplication at *t’ = 1* as their parental chromosome and the rest of the 2*N – s(2)* chromosomes will be randomly assigned a parental chromosome not carrying the duplication at *t’ = 1*. This process is continued until *t’ = k* – 1. Typically, because the average neutral fixation time is 4*N* generations, fixation trajectories will be larger than *k*. Thus, the aforementioned process will frequently exceed *k* generations.

Before the duplication reaches fixation, crossover might occur between chromosomes with a different number of blocks. In this case, irrespective of the underlying crossover model, the position of the crossover junction will be chosen from their shared length. Without loss of generality, the *daughter* chromosome will have the same number of blocks as its parental chromosome, regardless of the number of blocks of its parental chromosome’s partner. To ensure this, the daughter chromosome will inherit the region from the 5′ end until the crossover junction (including the junction) from its parental chromosome’s partner and the region from the junction until the 3′ end from its parental chromosome. At the end of every Era, the mutations present on every block in a random sample of *n* = 50 individuals (*i.e.*, 100 chromosomes) are recorded. Fixed derived mutations are erased from simulated chromosomes after being recorded (mutations on duplicated blocks are only erased if they are fixed in both blocks), which saves memory and running time. At the end of each simulation run, we will have recorded data every *k* generations.

### Variation measures

**Figure 2:**
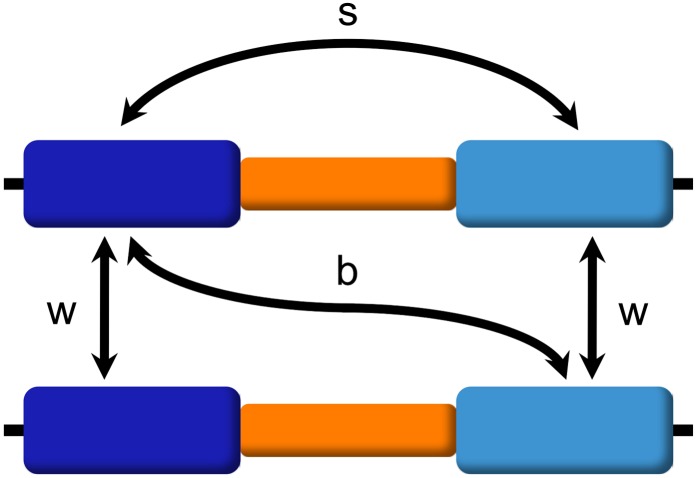
Variation measures. We measure nucleotide variation among segmental duplications as follows: (w) variation within duplicate blocks (at equilibrium, variation within the original and duplicated blocks will be the same); (b) variation between the original and duplicated blocks on different chromosomes; (s) variation between the original and duplicated blocks on the same chromosome. We use average pairwise differences (π) to measure all these types of variation (π_w_, π_b_, π_s_).

From the data recorded every Era, we extract site-frequency spectra and variation measurements such as the number of segregating sites (*S*) and the average number of pairwise differences (π). Although historically it makes sense to talk about sequence diversity and divergence when referring to duplicated regions of the genome, from here on and for the sake of simplicity, we will use the term *variation* to refer to both because we will measure both diversity and divergence by calculating π within and between copies. To compare the evolution of the duplicate blocks under different parameter values, we explore nucleotide variation with the following measures (Figure 2): variation within a duplicate block (π_w_) (at equilibrium, variation within the original block and within the duplicated block will be the same); variation between the original and duplicated blocks on different chromosomes (π_b_); and variation between the original and duplicated blocks on the same chromosome (π_s_). In addition, we also calculate the variation present within the single-copy block, which will serve as control.

**Figure 3:**
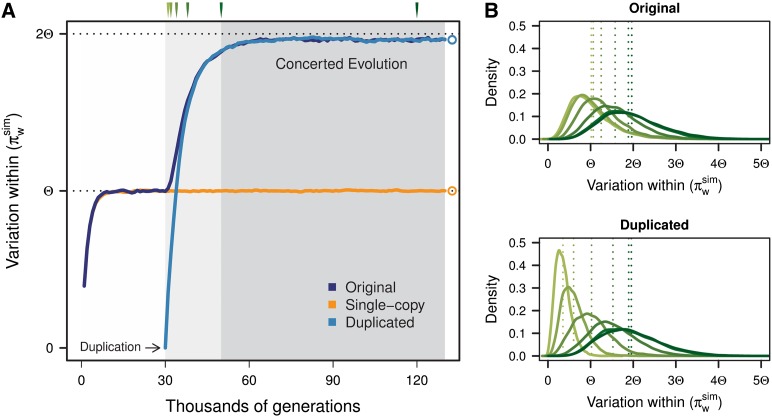
Changes in variation within blocks along simulations. Average results from 10,000 simulation runs are shown. (A) Dark blue, orange, and light blue curves correspond to the average pairwise differences found within the original, single-copy, and duplicated blocks, respectively. Gray-shaded areas correspond to the burn-in phase, structured phase, and concerted evolution phase. Duplication occurs at *t* = 30*N*. Although we depict the structured phase as ending at *t =* 50*N*, this is actually an arbitrary upper limit, because the neutral trajectory of the duplicated chromosomes and length of the structured phase is different every simulation. As expected, variation at equilibrium for the single-copy block is Θ = θ*L* = 4*N*μ*L*. The original and duplicated blocks attain higher variation (~1.95Θ) due to IGC activity among them. Parameters for this simulation are *N* = 1000, *k* = 1000, *L* = 5000, θ = 0.001, *C* = 0.5, *R* = 50, and λ = 100. (B) Distributions of π_w_^sim^ for the original (top) and duplicated (bottom) blocks at different times after the appearance of the duplication (*t* = 31*N*, 32*N*, 34*N*, 38*N*, 50*N*, and 120*N*) are colored in different shades of green.

Average values from 10,000 simulations of π within the original, the single-copy, and the duplicated blocks are represented in Figure 3A. During the burn-in phase, variation within the original and single-copy blocks reaches its neutral expectation π_w_ = Θ. Once the duplication appears (at *t = T_1_* + 1 *=* 30*N*), variation begins to increase in the duplicated block, and IGC activity begins between the original and the duplicated blocks. Distributions of the variation within the original and duplicated blocks at different time points are shown in Figure 3B.

### LD measures

To analyze how LD patterns vary with IGC rates and crossover models, we have devised a simple way of extracting LD measurements from independent simulation runs and of obtaining average values from them. To do so, we have binned every block in 100-bp windows. For every run, we analyze a sample of *n* = 50 individuals at *t* = *T_T_*, once the population has reached equilibrium. For every pair of windows *x* and *y*, we calculate *D*’ and *r^2^* between all possible pairs of mutations (one taken from *x* and the other from *y*). We then calculate the average value of all these measurements and thus obtain a value of *D*’ and *r^2^* for every pair of windows that is independent of the number of mutations that fall within a particular window in a particular run. We repeat this process for every simulation run and calculate the average values for every pair of windows. During the analysis of LD patterns, we will differentiate between LD within the duplicate regions and LD between paralogous windows of the original and duplicated blocks (from here on, we refer to the latter as *LD between duplicates*).

### Parameter values

In this paper, we concentrate on the effect of two parameters: the IGC rate (*C*), and the homologous crossover rate (*R*). Other parameters, such as the point mutation rate, only modify the scale and variance of our results. To allow for accurate comparisons with results from previous works, all our parameters are population scaled. Population size is an important parameter in so far as it does have an effect in populations undergoing natural selection. However, because we are exclusively considering a neutral scenario, we have fixed our population effective size to *N* = 1000. The range of parameters we have explored is based primarily on previous coalescent simulations (Innan 2003; Thornton 2007) and a literature survey selecting a range of empirical estimates from various species to guarantee that we are exploring realistic scenarios. In Table 1, the range of parameters we explore here is compared to those in previous works. Details about how they were selected can be found in File S1. In addition, File S2 shows how rates of IGC vary according to the distance between duplicated blocks. In this paper, we exclusively explore duplicate blocks of length *L* separated by a block of equal length. The Perl and R scripts to construct the LD plots, as well as the C++ forward simulator used throughout this study, are available upon request.

**Table 1:** Comparison between parameter ranges explored through simulations by Innan 2003, Thornton 2007, and this work

		Innan 2003	Thornton 2007	This Work
Parameters	Notation	Coalescent	Coalescent	Forward
Crossover rate per meiosis	R=4Nr	0, 1, 10	0, 100, 10000	0, 1, 10, 50, 100
Mutation rate per block per generation	Θ=4NμL	10	10	5
IGC rate per duplicated site per generation	C=4Nc	0.2, 1, 5	0, 1, 10	0.001, 0.01, 0.1, 0.5, 1, 5, 10, 50
Mean IGC tract length	λ	1 mut.	10, 500, 900	100
Number of loci	$n_{\text{2}}$	2	2, 5, 8	2
Effective population size	*N*			1000

IGC, interlocus gene conversion; mut., mutations per IGC event.

## Results

The action of gene conversion between the original and duplicated blocks increases the amount of variation found within each of them. Variation within blocks will attain an equilibrium value between Θ and 2Θ, which will depend on IGC and crossover rates. Figure 3 shows the case for *C* = 0.5, *R* = 50, and *N* = 1000, for which π_w_ ≈ 1.95Θ. IGC increases variation by transferring mutations that appear in one block onto the other block while at the same time preventing the fixation of different derived mutations in each block. However, a clear increment in variation is observed only for a limited range of IGC rates. On the one hand, very high IGC rates can homogenize copies to such a point that they remain nearly identical and maintain a level of variation similar to that of single-copy regions. On the other hand, very low IGC rates can increase variation up to 2Θ, but waiting times to reach this equilibrium can be extremely high (Nagylaki 1984).

Increased variation within duplicates caused by IGC activity has already been extensively studied and modeled for small and large multigene families. We have selected three different models (Ohta 1983; Innan 2002, 2003) to validate the results of our simulations on variation (File S3, Figure S1, and Figure S2) and on LD (File S4 and Figure S3). Given some disparity in nomenclature among this body of work, we have introduced a new notation differentiating models, which is explained in Table 2. After validation, we proceeded to examine in detail the interplay between IGC and crossover in determining levels and patterns of variation and LD in duplications. In order to do so, we analyzed three different crossover models: SCC, in which crossover is limited to the single-copy region; WRC, in which crossover can occur in the whole simulated region; and HSC, in which crossover happens at a previously defined hotspot or hotspots.

**Table 2:** Nomenclature used throughout this work to distinguish between different types of variation, and allowing for comparison between theoretical models by Ohta (1983) and Innan (2002, 2003)

Type of Variation	Ohta 1983	Innan 2002	Innan 2003	This Work
	Model A	Model B	Model C	Simulations
Variation within blocks	$\pi_{w}^{A}=\left(\text{1}-f\right)L$	$\pi_{w}^{B}=E\left(h_{w}\right)L$	$\pi_{w}^{C}=E\left(\pi_{w}\right)$	$\pi_{w}^{sim}$
Variation between blocks on different chromosomes	$\pi_{b}^{A}=\left(\text{1}-c_{\text{2}}\right)L$	$\pi_{b}^{B}=E\left(h_{b}\right)L$	$\pi_{b}^{C}=E\left(\pi_{b}\right)$	$\pi_{b}^{sim}$
Variation between blocks on the same chromosome	$\pi_{s}^{A}=\left(\text{1}-c_{\text{1}}\right)L$			$\pi_{s}^{sim}$

### SCC model: single-copy crossover

Under this model, crossover occurs only in the single-copy region between duplicates. The levels of variation found for the SCC model fall directly on the theoretical predictions provided by Ohta (1983) and Innan (2002, 2003) with a very high degree of accuracy (for a detailed description and comparison between these models see File S3, File S5, Figure S1, Figure S2, and Figure S4). Under the infinite-site model, variation within a duplicate at equilibrium is negatively correlated with IGC rate and positively correlated with crossover rates. The explanation for the latter is that the higher the crossover rate, the higher the probability for two duplicate blocks that have already undergone IGC to become separated, thus allowing for another IGC event to effectively transfer different variants from one block to another.

**Figure 4:**
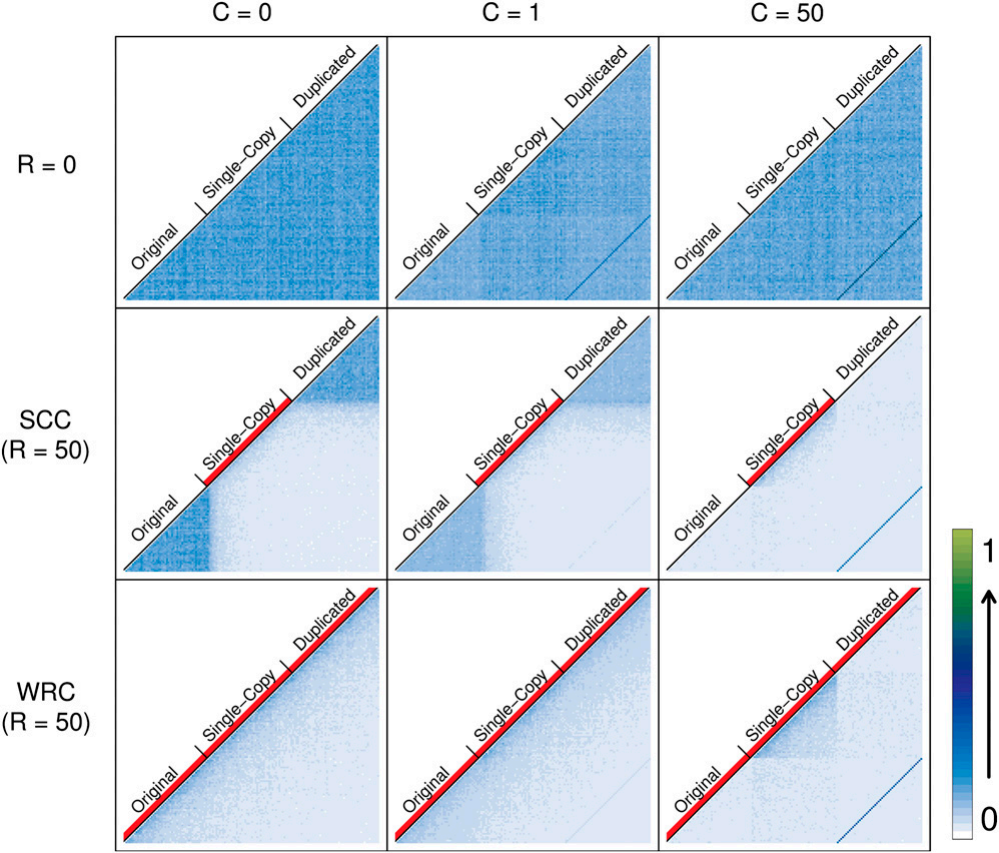
LD patterns under different crossover models. Average values for 1000 simulations are shown. LD between each pair of windows along the sequence (D′) is coded with a number between 0 and 1 and represented with a color (from white to dark blue to light green). Three different IGC rates (0, 1, and 50) are represented in columns and three different crossover conditions are shown in rows: no crossover, SCC (*R* = 50), and WRC (*R* = 50). The red lines below the names identifying each block show regions undergoing crossover. In the first row, where no crossover is acting, a dark blue diagonal line appears when IGC is active (and increasing with IGC rate) representing LD between paralogous windows of duplicate blocks. LD within the duplicate region is high when no IGC is acting and when IGC is high (*C* = 50) but decreases with a medium IGC rate. LD between duplicates (dark blue diagonal line) decreases when crossover is active on the single-copy region (SCC model, row 2) and on the whole region (WRC model, row 3) with respect to *R* = 0. As expected, crossover breaks LD blocks in the regions where it is acting. IGC also breaks LD blocks within duplicates if crossover is active (both in the SCC model and in the WRC model).

Figure 4 and Figure S5 show LD patterns (D′ and r^2^, respectively) for different crossover models and IGC rates. Row 1 shows the case for *R* = 0 and provides a null expectation for the effect of IGC in the absence of crossover: high LD between duplicates (see *Materials and Methods*), represented by a diagonal dark blue line. Under the SCC model (row 2), we observe that in the presence of crossover, the pattern of LD in regions undergoing IGC changes considerably. LD within the single-copy block will be low due to recurrent crossover within it, relative to the higher levels of LD in the original and duplicated blocks where no crossover occurs. However, this only holds for low IGC rates, because moderate-to-high IGC between duplicates also breaks down LD within them. Furthermore, LD between duplicates will always increase with increasing IGC rate but will decrease with the rate of crossover.

In the absence of crossover, moderate IGC decreases the amount of LD within duplicate blocks. This very weak effect is expected because IGC breaks linkage within blocks by transferring new variants from the other duplicate. High IGC does not break this linkage since variants are always exchanged between duplicates on the same chromosome (Figure 4, row 1). However, when crossover is active, the effectiveness of IGC in breaking LD blocks increases dramatically and correlates positively with IGC rate and crossover rates (data not shown for different crossover rates) (Figure 4, row 2).

### WRC model: whole-region crossover

**Figure 5:**
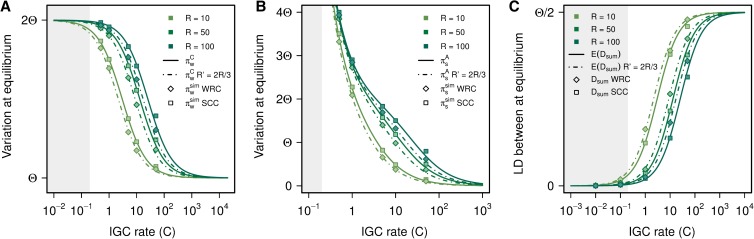
Comparison between data from simulations under the SCC model and the WRC model. Results are shown for (A) π_w_^C^, (B) π_s_^A^, and (C) E(D_sum_). Continuous lines are based on the SCC model and are shown for different crossover rates (*R* = 10, 50, and 100). As expected, results from SCC simulations are in very good agreement with theoretical expectations. Results from WRC simulations agree with theoretical expectations (discontinuous lines) for *R*’ = (2/3)*R*, showing that allowing crossover to occur in the duplicate regions has an effect identical to that of effectively reducing the crossover rate by one third on the SCC model. Although we have not implemented any IGC rate dependence on sequence similarity between duplicates, according to Walsh (1987) IGC rates *C* > 0.2 would ensure the prevalence of stable concerted evolution at least temporarily in the face of genetic drift (see File S1). The shaded area indicates the region that lies beyond this threshold, where both theoretical predictions and results from our simulations might not be biologically realistic.

Because it is likely that crossover is not restricted to the single-copy region between duplications, we explore the interplay between IGC and crossover by allowing crossover to extend over the whole simulated region. Allowing crossover to overlap with regions subject to IGC events decreases the expected within-block variation for all IGC and crossover rates (Figure 5). Points for the WRC model fall on curves corresponding to *R*’ = (2/3)*R*. That is, given that we are simulating three blocks of equal length, allowing crossover to occur on the whole region has an effect on variation equivalent to decreasing the crossover rate by one third under the SCC model (for a detailed explanation, see File S6). This effect is observed for variation within blocks (Figure 5A), variation between blocks (Figure 5B), and LD between duplicates (Figure 5C).

Row 3 of Figure 4 shows the pattern of LD under the WRC model. As expected, LD blocks present within the original and duplicated blocks under the SCC model disappear when allowing crossover to occur within duplicate blocks. As the rate of IGC increases, LD within duplicate blocks decreases, while LD levels and patterns remain constant within the single-copy block, where no IGC is acting. In accordance with the reduction of the strength of crossover depicted in Figure 5, there is an increase of LD between duplicates under the WRC model compared to the SCC model.

### HSC model: hotspot crossover

**Figure 6:**
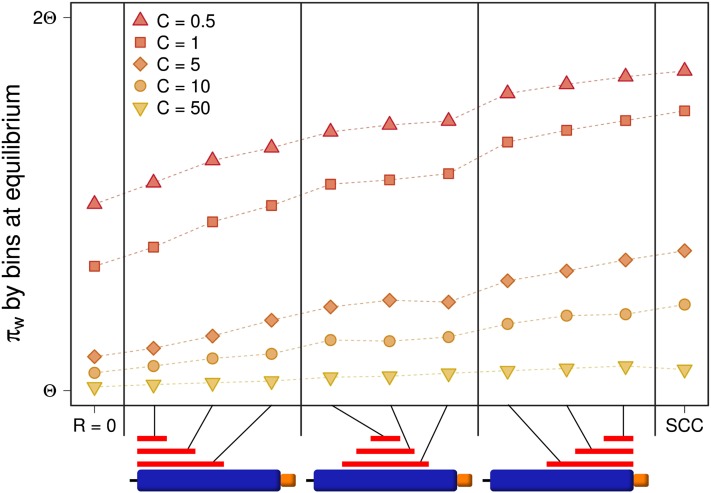
Variation within a duplicate block under different crossover conditions. The plot shows variation within duplicates under the HSC model. Each point corresponds to the average equilibrium value over 1000 simulation runs. Columns indicate different hotspot locations on the original block (illustrated by the red lines in the diagram). Expected values (π_w_^C^) for *R* = 0 and for the SCC model (*R* = 10) are shown to the left and to the right of the plot respectively. Regardless of the width and localization of the hotspot, variation within duplicates is decreased in comparison with the SCC model but increased with respect to *R* = 0. Hotspots located the furthest away from the duplicated block (to the left) have the strongest effect in lowering the amount of variation, while those localized closest to the duplicated block still lower the variation but to a lesser degree (to the right). Hotspots centered in the original block have an intermediate effect.

Finally, the HSC model considers that crossover occurs in short and specific segments of the sequence. We first explore the effect of the presence of a crossover hotspot in one of the copies (in this case, the original copy). We show that if the hotspot is located toward the left of the whole simulated region (this is, furthest away from the single-copy region in our model) the average variation within the whole original block is significantly reduced compared with that found under the SCC model (Figure 6). In contrast, if the hotspot is located adjacent to the single-copy region, variation is reduced to a much lesser extent. Simulations with centered hotspots reach intermediate levels of variation. Minimum and maximum variation are achieved for *R* = 0 and under the SCC model, respectively. The average variation corresponding to any hotspot location falls between these two extreme values for a given IGC rate.

**Figure 7:**
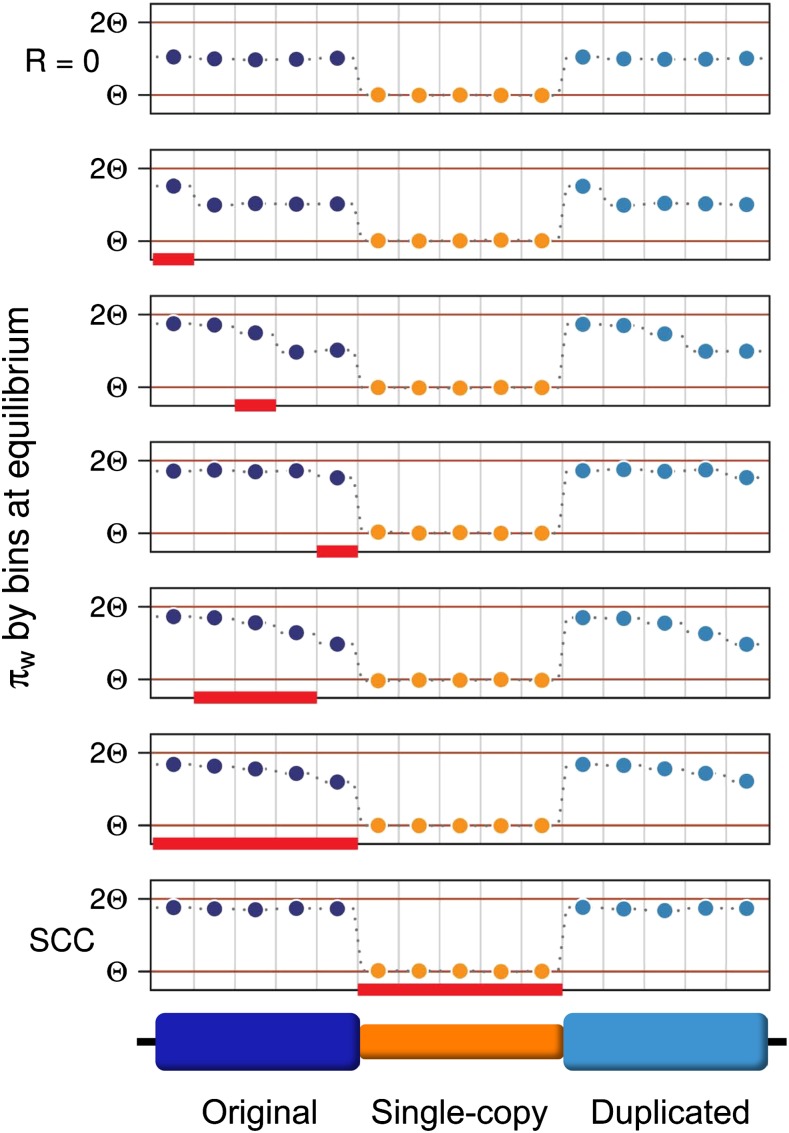
Distribution of variation along the sequence under different crossover conditions. Comparison between variation along the sequence when *R* = 0 (top row) and when crossover occurs (*R* = 10) on different conditions. Red rectangles indicate the regions undergoing crossover. In the bottom, variation along the sequence on SCC model is shown. Plots in the middle show different HSC model cases (different locations and lengths of the crossover hotspot region). Circles correspond to average pairwise differences calculated by bins within the original, single-copy and duplicated blocks, respectively. Each block is divided into five bins. Bins to the left of the hotspot have an amount of variation similar to that found under the SCC model whereas those to the right have a variation level equivalent to that of a model with no crossover (*R* = 0). Bins within the hotspot have intermediate levels of variation, which are lower for bins that are closer to the single-copy region. Additionally, original and duplicated blocks have identical (non-symmetrical) patterns of variation within them. This figure is for *C* = 0.5. Equivalent results are attained for greater values of *C*.

To better understand the reasons behind this decrease in variation, we calculated nucleotide variation in 1-kb bins and analyzed results for several hotspot locations. Results are depicted in Figure 7. We observe that IGC between paralogous regions makes the variation pattern identical for original and duplicated blocks. We also observe that the HSC model affects variation levels on each bin depending on their position relative to the hotspot. Bins to the left of the hotspot have the same levels of variation as they have under the SCC model; bins to the right of the hotspot have the same level of variation as they have under null crossover; and bins within the hotspot have intermediate values of variation between these two extremes. In the latter case, the fall in variation is gradual (from left to right) and the variation on each bin within the hotspot depends on the hotspot length and the position of the bin within the hotspot. These results show that hotspot location within regions undergoing constant IGC can affect levels of variation within duplicate regions.

Given the evidence that crossover hotspot location is dependent on sequence motifs, at least in the case of humans (Myers *et al.* 2008), it would make sense for hotspots to be present in both duplicates. Since the effect of crossover is symmetrical with respect to the center of the simulated region while IGC acts between paralogous regions, the decrease in variation within duplicate blocks observed under the HSC model with one hotspot is not maintained with two hotspots (Figure S6 and Figure S7).

**Figure 8:**
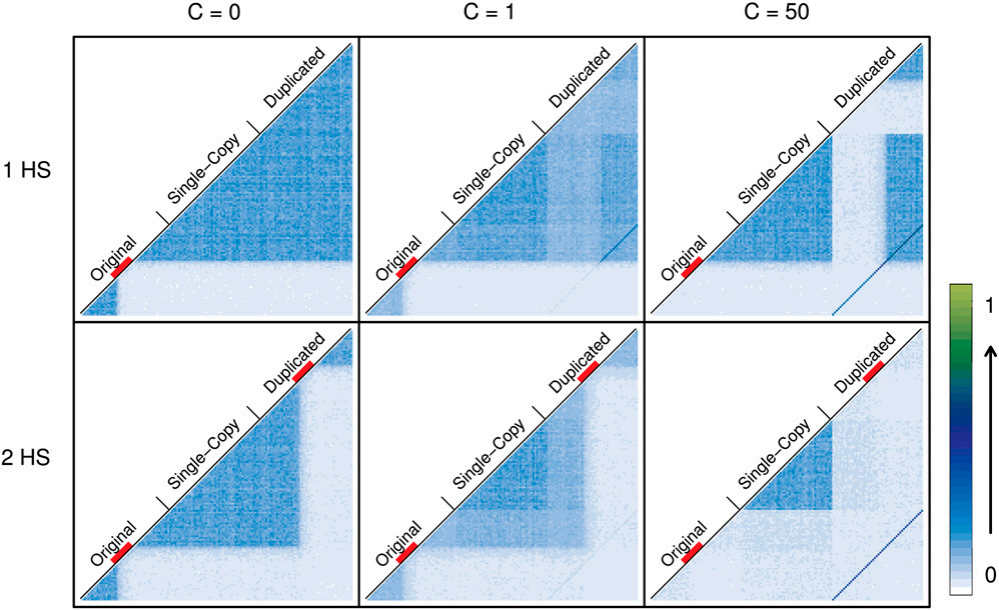
Comparison of LD between scenarios with one or two crossover hotspots. Average values for 1000 simulations are shown. LD between each pair of windows along the sequence (D′) is coded with a number between 0 and 1 and represented with a color (from white to dark blue to light green). Three different IGC rates (0, 1, and 50) are represented in columns. Rows show the effect of crossover (*R* = 50) located in one specific region of the original block or in both the original and duplicated blocks (in paralogous regions). The red line below the names identifying each block shows regions undergoing crossover. In the first column, the effect of crossover delimiting LD blocks is clear. When IGC is active (in the second and third column), a complex pattern of LD appears along the sequence together with the dark blue diagonal line representing LD between duplicate regions (stronger when no crossover is acting between paralogous windows of the duplicate blocks). With the presence of one hotspot on the original block, there are paralogous windows to the right of each duplicate between which there is no crossover and, thus, IGC has lower power to break LD in these fragments. In the case of two hotspots, this situation disappears and the combination of crossover and IGC breaks LD within duplicate blocks.

Figure 8 and Figure S8 show LD patterns (D′ and r^2^, respectively) under the HSC model with one and two hotspots for different IGC rates. As expected, if *C* = 0, highly identifiable LD blocks form to the left and right of the hotspot or hotspots. Contrary to the SCC model, LD blocks extend from the original to the duplicated block. For *C* > 0 and a single hotspot, a complex pattern appears driven by the breakdown of LD blocks within duplicates. As we have observed for the SCC model and the WRC model, LD blocks within duplicates break down strongly with IGC only if crossover is active between them. Under the HSC model with a single hotspot, LD breaks down to the left of the hotspot and in the corresponding paralogous region in the duplicated block; this breakdown is unnoticeable, although present, to the right of the hotspot for *C* = 1. LD between duplicates is greater to the right of the hotspot location than to the left of the hotspot location since crossover is acting between paralogous regions only to the left of the hotspot.

Under the HSC model with two hotspots, crossover generates a symmetrical pattern of LD blocks in both duplicates. As a consequence, when IGC is active between paralogous regions, it breaks down LD not only in the external regions with respect to the hotspots but also in their corresponding paralogous (internal) regions, resulting in a complete breakdown of the LD along the duplicated blocks.

### Overview

We have described the effects of IGC under different crossover models and highlighted the importance of the distribution of crossover junctions in the attainment of variation within and between duplicates. As we have shown, crossover between paralogous regions provides the opportunity for IGC to effectively transfer new variants between paralogous regions and for IGC to break LD within duplicates more efficiently. If crossover is not acting between copies, variation will be lower and LD will be higher within paralogous regions for the same IGC rates. In the case of the HSC model, we can find both situations in the same duplicated region, creating a complex pattern of nucleotide variation and LD along the duplicates. We have summarized the different possible scenarios in Table 3.

**Table 3:** Summary of variation and LD measures under different IGC and crossover conditions

Crossover Between Paralogous Regions	IGC Rate		
	*C* ≈ 0	*C* ≈ 1	*C* ≈ 100
*R* ≈ 0	π_w_ ≈ Θ (min)	π_w_ ≈ intermediate	π_w_ ≈ Θ (min)
	π_b_ ≈ 2ΘT_d_ (max)	π_b_ = intermediate	π_b_ ≈ Θ (min)
	π_s_ ≈ 2ΘT_d_ (max)	π_s_ = low	π_s_ ≈ 0 (min)
	LD_w_ ≈ high	LD_w_ = intermediate	LD_w_ ≈ high
	LD_b_ ≈ high	LD_b_ = high	LD_b_ ≈ 1 (max)
*SCC (R* ≈ 50)	π_w_ ≈ Θ (min)	π_w_ ≈ 2Θ (max)	π_w_ ≈ Θ (min)
	π_b_ ≈ 2Θ*T*_d_ (max)	π_b_ = intermediate	π_b_ ≈ Θ (min)
	π_s_ ≈ 2Θ*T*_d_ (max)	π_s_ = intermediate	π_s_ ≈ 0 (min)
	LD_w_ ≈ high	LD_w_ = intermediate	LD_w_ ≈ low
	LD_b_ ≈ 0 (min)	LD_b_ = intermediate	LD_b_ ≈ high

*T*_d_ is time in generations since duplication; LD, linkage disequilibrium; IGC, interlocus gene conversion; SCC, single-copy crossover.

## Discussion

The complexity behind the interplay between IGC and crossover was already evident from theoretical models of the effect IGC and crossover on levels of variation within and between duplicates under neutrality (Ohta 1982, 1983; Nagylaki 1984; Innan 2002, 2003). However, extending its application not only to duplicated genes but also to SDs, which can span large areas of the genome and are not necessarily in tandem, demands the incorporation of more realistic crossover models. To tackle SD evolution, forward-time simulators promise to be an efficient tool for intense exploration of broad ranges of parameter values. Here, we have presented a first glimpse at what forward-time simulations can offer, limiting our analysis to neutral evolution and thus, providing a null-model for future scans of areas under selective pressure within SDs.

Increased variation within duplicates undergoing IGC is an accepted phenomenon (Baltimore 1981; Ohta 1982, 1983; Nagylaki 1984; Walsh 1987; Innan 2002, 2003; Thornton 2007; Ohta 2010; Teshima and Innan 2012) and it has been observed in humans (Bosch *et al.* 2004; Hallast *et al.* 2005) as well as in other species (Nielsen *et al.* 2003; Rane *et al.* 2010; Willett 2013). In this study, we have shown that the extent to which variation within duplicates is increased due to IGC is highly dependent upon the distribution of crossover junctions. In the case of crossover hotspots, if they happen to fall inside duplications, the distribution of variation within these regions might be altered, with a strong dependence on the hotspot location. This complex pattern of variation might affect the fate of SDs and could be important to take into account when calculating IGC rates. The implications of increased variation on the evolutionary fate of SDs are largely unexplored. For instance, high variation might increase the possibility of fixation of compensatory mutations (Plotnikova *et al.* 2007) and afford more opportunities for natural selection to act upon standing variation (Katju *et al.* 2008). This will not be independent of the underlying recombination scenario under which certain levels of variability are achieved.

Abundant IGC between duplicates may facilitate parallel selective sweeps if increased gene dosage is positively selected (Hanikenne *et al.* 2013). IGC is additionally thought to be one of the mechanisms responsible for the elimination of deleterious mutations in polyploid asexual genetic systems (Khakhlova and Bock 2006) and in the human Y chromosome (Rozen *et al.* 2003; Marais *et al.* 2010), allowing them to escape Muller’s ratchet. Alternatively, deleterious effects of IGC also have been reported; Casola *et al.* (2012) demonstrated that the introduction of deleterious alleles via IGC has happened in at least 1% of human genes and identified thousands of potentially deleterious mutations that could be disease-causing if they were to have the same fate.

Another interesting result concerns LD. We have observed that the relationship between LD and IGC rate is nonlinear because both very low and very high IGC rates will produce high LD within duplicates, whereas intermediate IGC rates produce intermediate levels of LD. This effect cannot be observed if crossover is active between duplicates because the interplay between IGC and crossover drastically increases the breakdown of LD within duplicates. We have also analyzed the levels of LD between duplicates and found that they have a positive correlation with IGC rates and a negative correlation with crossover rates.

Gene conversion is already known to affect patterns of LD and is considered to be the main cause of LD breakdown over short distances (Andolfatto and Nordborg 1998; Ardlie *et al.* 2001; Frisse *et al.* 2001; Plagnol *et al.* 2006). However, this observation is restricted to gene conversion within the same loci on different chromosomes, referred to as intralocus gene conversion, interallelic gene conversion, or simply gene conversion. It has been suggested that similar molecular mechanisms might be behind both intralocus and ICG (Jeffreys and May 2004). However, even if a similar mechanism could be illustrated (Hastings 2010), it does not imply that their effects on variation or on patterns of LD should be equal. In this work, we have exclusively dealt with gene conversion between different loci (IGC) and have analyzed, through simulations, the effect of IGC on LD patterns (D’ and r^2^) not only within but also between duplicates. Patterns of LD inside gene families have received some attention (Hallast *et al.* 2005), but the causes for the appearance and maintenance of these patterns have not been explored thoroughly. We have demonstrated that in the case of IGC, the interplay with crossover is crucial to determine the levels of LD. We stressed the importance of differentiating between LD within and LD between duplicates because they do not respond in the same way to the action of IGC and crossover and might prove to be useful in ascertaining biases such as donor-acceptor bias.

Under neutrality, there are at least two possible mechanisms by means of which different levels of variation and LD can be attained along a duplicated sequence: nonhomogeneous distribution of IGC events and nonhomogeneous distribution of crossover junctions. IGC rates are known to be dependent on sequence similarity between paralogous regions. We suspect that setting minimum identity thresholds for IGC to occur (see File S1) may cause patterning inside SDs. Our simulator incorporates the possibility of setting these thresholds but the effects of these are beyond the scope of this paper. Patterning could also be caused by the nonhomogeneous distribution of crossover junctions that we have explored here. All crossover models share a common characteristic: given a homogeneous IGC rate, crossover is effective in increasing variation and in breaking LD within duplicates only if it is acting between paralogous regions. Of course, crossover does not play an “active” role in these effects; it only sets the ground for IGC to cause them.

The presence of crossover hotspots within SDs is of special relevance given the asymmetrical patterns it can generate within them. If a crossover hotspot is located inside a copy of the duplication but not in its paralog, increased variation might only be found in the region that is separated from its paralog by the hotspot and not in the rest of the SD. This asymmetry might tend to disappear via IGC (if it were caused by a sequence motif), either by the elimination of the hotspot or its transfer to the duplicate. For a short period of time, however, this asymmetry might by decisive in determining the evolutionary fate of genes located in different regions within the SDs.

The analysis that we presented here covers a broad range of IGC rates that spans the observed ranges in a wide variety of species. The applicability of our results to real data depends on the extent to which the measurement of IGC rates between duplicates is accurate. As evidenced by Mansai and Innan (2010) some methods to detect IGC rates, such as GENECONV (Sawyer 1989), can underestimate IGC rates if they are very high. New methods are still being developed (*e.g.*, Dumont and Eichler 2013). We believe that for tandem duplications, the LD measurement method that we have implemented here would be able to detect rates of IGC between duplicates, especially if they are high. A formal comparison between our method and others would be an interesting focus of further research.

Given the relevance of IGC between SDs in primate and human recent evolution (Fawcett and Innan 2013) and their likely role in adaptation (Bailey and Eichler 2006; Han *et al.* 2009) we have provided a testing ground by analyzing the neutral scenario. Exploring the effects of natural selection on the evolution of duplicated regions of the genome would be a natural next step and an important and surely interesting endeavor.

## Supplementary Material

Supporting Information
