# Supplementary material for: Interplay of Interlocus Gene Conversion and Crossover in Segmental Duplications Under a Neutral Scenario
Source: G3 (Bethesda). 2014 Jun 6;4(8):1479–89. doi: 10.1534/g3.114.012435 (PMC4132178; doi:10.1534/g3.114.012435)
Supplement: Supporting Information [file supp_4_8_1479__index.html]

Interplay of Interlocus Gene Conversion and Crossover in Segmental Duplications Under a Neutral Scenario — Supporting Information 

# Interplay of Interlocus Gene Conversion and Crossover in Segmental Duplications Under a Neutral Scenario

## Supporting Information for Hartasánchez *et al.*, 2014

**Files in this Data Supplement:**

- Supporting Information - Files S1-S6, Figures S1-S8, and Additional References (PDF, 1 MB)
- File S1 - Parameter Values (PDF, 139 KB)
- File S2 - Dependence of IGC rate on distance between duplicates. (PDF, 122 KB)
- File S3 - Validation on variation. (PDF, 159 KB)
- File S4 - Validation on linkage disequilibrium. (PDF, 151 KB)
- File S5 - Comparison between theoretical expectations of variation between duplicates. (PDF, 151 KB)
- File S6 - The effect on variability of allowing crossover in the whole simulated region. (PDF, 122 KB)
- Figure S1 - Comparison of main measures of variation. (PDF, 173 KB)
- Figure S2 - Comparison of variation within blocks between models. (PDF, 210 KB)
- Figure S3 - LD between duplicates. (PDF, 166 KB)
- Figure S4 - Comparison between different theoretical expectations of variation between duplicates. (PDF, 288 KB)
- Figure S5 - LD along the sequence under different crossover models. (PDF, 282 KB)
- Figure S6 - Variation within a duplicate block under different cases of HSC with two hotspots. (PDF, 472 KB)
- Figure S7 - Distribution of variation along the simulated sequence under different two-hotspot HSC cases. (PDF, 264 KB)
- Figure S8 - Comparison of LD along the sequence between models with one or two crossover hotspots. (PDF, 451 KB)
