# Supplementary material for: Interplay of Interlocus Gene Conversion and Crossover in Segmental Duplications Under a Neutral Scenario
Source: G3 (Bethesda). 2014 Jun 6;4(8):1479–89. doi: 10.1534/g3.114.012435 (PMC4132178; doi:10.1534/g3.114.012435)
Supplement: Supporting Information [file supp_g3.114.012435_012435SI.pdf]

G3·Genes | Genomes | Genetics

Interplay of Interlocus Gene Conversion and Crossover in Segmental Duplications under a Neutral Scenario

Diego A. Hartasánchez*, Oriol Vallès-Codina*, Marina Brasó-Vives*, Arcadi Navarro* § † ‡

*Institute of Evolutionary Biology (Universitat Pompeu Fabra – CSIC), PRBB, Barcelona, Catalonia, Spain, 08003.
§National Institute for Bioinformatics (INB), Barcelona, Catalonia, Spain.
†Institució Catalana de Recerca i Estudis Avançats (ICREA), Barcelona, Catalonia, Spain.
‡Centre for Genomic Regulation (CRG). Barcelona, Catalonia, Spain, 08003.

DOI: 10.1534/g3.114.012435

File S1

Parameter Values

The range of parameters we have explored is based primarily on previous coalescent simulations by Innan (2003) and Thornton (2007). The parameter values explored here are compared to theirs in Table 1 in the main text. Here we will also consider different crossover models.

IGC rates

The rate of IGC has been shown to depend on a series of factors, reviewed by Chen *et al*. (2007) and discussed in detail by Mansai *et al*. (2011), who contrast empirical and evolutionary approaches to measure IGC rates; by Benvoy and Drouin (2009), who analyze IGC between human protein coding genes; by McGrath *et al.* (2009), who do so for human, macaque, mouse and rat; and Casola *et al.* (2010) in the genus *Drosophila*. Here we will briefly mention these factors in order to justify our choice of parameters, and also to clarify that IGC models like the one we implement here are still far from being close to reality, in part because of a lack of consistency between different studies on IGC rates. In our simulations, we test IGC rates of C from 0.001 to 100 in populations of N = 1000. Therefore, IGC rates per site per generation (c) range from 2.5×10^{-7} to 2.5×10^{-2}.

Organism: Genome architecture generates differences in IGC rate between organisms, with estimates ranging, for instance, from $\sim 10^{-10}$ to $\sim 10^{-3}$ in *S. cerevisiae* (cited in Mansai *et al.* 2011) to $\sim 10^{-4}$ to $\sim 10^{-3}$ in humans (Chen *et al.* 2007).

Meiosis vs. mitosis: IGC rate has been reported to be from ~15 to ~300 times higher in meiosis than in mitosis in yeast (cited in Mansai *et al.* 2011).

Intrachromosomal vs. interchromosomal: In humans, intrachromosomal IGC events are ~5 times more frequent than their interchromosomal equivalent (Benovoy and Drouin 2009). A similar relationship has been found in mammals (McGrath *et al*. 2009), yeast (Petes and Hill 1988) and *C. elegans* (Semple and Wolfe 1999). Here we exclusively simulate meiotic intrachromosomal IGC events.

Distance between duplicates: The rate of IGC seems to be negatively correlated with distance between the duplicated regions involved in intrachromosomal IGC events (Lichten and Haber 1989; Semple and Wolfe 1999; Benovoy and Drouin 2009; Aleshin and Zhi 2010; Casola *et al.* 2010). This could in fact be a consequence of neighboring paralogs being more recent and therefore less divergent on average, implying higher IGC rates (McGrath *et al.* 2009). In humans, the median distance between intrachromosomal converted genes is $7.8 \times 10^{4} \pm 2.88 \times 10^{7}$ bp and more than 50% of conversions occur between genes that are less than 10^{4} bp apart (Benovoy and Drouin 2009). For more details, see File S2.

Orientation: Although there is an excess of IGC events between genes with the same orientation (Semple and Wolfe 1999; Benovoy and Drouin 2009; Aleshin and Zhi 2010), it might be the consequence of an excess of close-by duplicated genes (less than 50 kb from each other) in a head-to-tail orientation (McGrath *et al.* 2009).

Sequence similarity: IGC rates seem to be positively correlated with DNA sequence similarity between converted pairs of genes in unconverted regions (Semple and Wolfe 1999) with most conversions occurring for similarity levels above 95% (Chen *et al*. 2007; Sudmant *et al*. 2010). The strength of this correlation might be biased because the most commonly used software to detect conversions (GENECONV by Sawyer *et al.* 1989) lacks power when divergence between duplicates is very low (McGrath e*t al*. 2009). The minimal degree of identity necessary for IGC to occur or *minimal efficient sequence homology* (MESH) is ~92% (Chen *et al.* 2010). Walsh (1987) examined models under which IGC frequencies decreased due to sequence divergence between duplicates. Considering a model where IGC rates decrease linearly with sequence divergence and exclusively through accumulation of point mutations, Walsh states that if $2\mu/c << 0.1$ genes never escape IGC. Setting $\theta = 0.001$ and considering $2\theta/C = 0.01$ as our threshold, this would imply that IGC rates $C > 0.2$ would ensure stable concerted evolution. In this work, we consider IGC to act without any restriction on sequence similarity but concentrate our analyses on IGC rates $C > 0.2$.

Sequence identity: Additional to the overall level of divergence between duplicated regions, there appears to be a *minimal efficient processing segment* (MEPS) (Shen and Huang 1986); for there to be effective gene conversion, a segment of 100% identity needs to be adjacent to the gene conversion tract. Measurements of MEPS in mammalian cells fall around 200 bp: between 200 and 295 bp (Liskay *et al*. 1987) and between 134 and 232 bp (Waldman and Liskay 1988). MEPS for humans has been estimated to lie between 337 and 456 bp (Reiter *et al*. 1998), although they can be as short as 40 bp for *Alu* repeats (Aleshin and Zhi 2010). In this study, we allow IGC to act irrespective of the existence of a MEPS. The inclusion of MESH and MEPS in future studies would be of relevance, particularly in the case of low IGC rates.

Local crossover rates: We know that IGC is associated with crossover of flanking sequences (Lichten *et al.* 1987), and it would therefore make sense for local IGC rate to be dependent on local crossover rates. Significant (Benovoy and Drouin 2009) and non-significant (McGrath *et al*. 2009) correlations between frequency of IGC events and local rates of recombination have been reported.

Gene conversion tract lengths

Although most available estimates on gene conversion tract lengths are from intralocus gene conversion, the molecular mechanism involved in both intralocus and interlocus (IGC) gene conversion is generally considered to be the same (Jeffreys and May 2004). Estimates for average tract lengths are extracted from measurements of minimum and maximum values and range from 55-290 bp for the average gene conversion tract length in human hotspot DNA3 (Jeffreys and May 2004), to 210 bp in human genes shared with macaque, mouse and rat (McGrath *et al*. 2009), to 31 bp (assuming a gene conversion event to have occurred if at least two variant sites have been converted) for two human endogenous retroviral (HERV) sequences located on chromosome Y (Bosch *et al.* 2004). For human protein coding genes, a maximum of 10 bp and a maximum of 6011 bp have been detected (Benovoy and Drouin 2009), but the latter might be the case of several overlapping gene conversions, since according to Chen *et al*. (2007), single gene conversion events rarely exceed 1 kb in humans.

There is evidence suggesting that gene conversion tract lengths depend on the level of sequence divergence between interacting sequences (Lukacsovish and Waldman 1999). Considering the maximum sequence similarity between the 100 nucleotides upstream and downstream of the gene conversion event, Benovoy and Drouin (2009) show that gene conversion events shorter than 1 kb in length can occur with flanking regions with sequence similarity as low as 23.8%, whereas larger than 1 kb gene conversion events only occur is sequence similarity is above 89%. This finding might imply a cutoff in the gene conversion machinery if sequence similarity happens to fall (Benovoy and Drouin 2009), although, to the best of our knowledge, this has not been formally tested.

Average IGC tract length estimates for other organisms such as yeast and rodents are also close to 100 bp (Mansai *et al.* 2011). In simulations, common average conversion tract lengths used range from 100 to 1000 bp (Thornton 2007; Mansai and Innan 2010). Throughout this work the average IGC tract length is $\lambda = 100$ bp.

Recombination

In 1996, Collins *et al*. established a sex-averaged figure of 0.88 Mb equal to 1 cM in humans (Collins *et al.* 1996). Since then, an approximation of 1 cM/Mb (0.01 crossovers per generation per Mb) has been adopted as a consensus figure that results in a per nucleotide per generation homologous recombination rate of 1×10^{-8}. Since they are genome-wide averages, these figures include crossover activity in hotspots. If we were to consider that there are no hotspots within the region we simulate, it would be better to consider the background crossover rate, estimated to be of 0.1 cM/Mb that is, 10^{-9} recombination events per nucleotide per generation (McVean *et al*. 2004).

We test crossover rates of R = 0, 1, 10, 50, and 100, in populations of N = 1000. Therefore, crossover rates per chromosome per generation (r) vary from 2.5×10^{-4} to 2.5×10^{-2} (evidently, excluding the case of r = 0). In the case of SCC (and L = 5000), these correspond to per-site crossover rates from 5×10^{-8} to 5×10^{-6}; for WRC, from 1.7×10^{-8} to 1.7×10^{-6}, and for HSC with 1 kb hotspots, from 2.5×10^{-7} to 2.5×10^{-5}.

In this paper, we ignore homologous recombination resolution by intralocus gene conversion, we exclusively simulate crossover (meaning exchange of tails) to one side of the crossover junction.

File S2

Dependence of IGC rate on distance between duplicates

There is evidence from many species that the frequency of intrachromosomal IGC events increases as the distance between the implicated genes decreases [humans (Benovoy and Drouin 2009; McGrath *et al*. 2009), mouse (Ezawa *et al*. 2006; McGrath *et al*. 2009), *Drosophila* (Casola *et al.* 2010), yeast (Goldman and Lichten 1996) and *C. elegans* (Semple and Wolfe 1999)]. However, this excess of IGC activity is attributed to different causes by different authors. Benovoy and Drouin (2009) attribute it to the fact that most duplicated functional genes lie in close vicinity to each other. They argue that the majority of gene family members are within 10 kbp of each other whereas most unrelated genes lay further away (Benovoy and Drouin 2009). Interestingly, McGrath and collaborators argue that the inverse proportionality between physical distance and frequency of IGC might just be a consequence of less divergence among closely placed duplications and not a direct consequence of the physical distance between them. Since many duplications arise through NAHR, neighboring duplicates are likely to have a more recent origin and thus be less divergent between them. Discriminating between these confounding factors is complicated since an IGC event directly affects divergence, and IGCs between highly similar sequences are difficult to detect (Mansai and Innan, 2010). In mammals, results show that once divergence between duplicates is accounted for, there is no significant relationship between physical distance and IGC rates (McGrath *et al*., 2009).

Regardless of its underlying cause, there is a negative correlation between IGC rates and physical distance between duplicates. It is clear that increasing the distance between two points along a chromosome will tend to increase the chance for there to be a crossover between them. Therefore, crossover and IGC rates have, respectively, a positive and negative correlation with physical distance between duplicates. Since equilibrium diversity within each block is also positively correlated with crossover rates between blocks, but negatively correlated with IGC rates, we can expect, in principle, a positive correlation between diversity in duplicated blocks and the distance between them. Closely located duplicates will be expected to have higher identity and lower diversity than duplicates further away from each other.

File S3

Validation on variation

Increased variation due to the effect of IGC has been extensively studied and modeled for small and large multigene families. We have selected three different models (Ohta 1983; Innan 2002, 2003) in order to validate the results of our simulations.

Model A (Ohta 1983): Ohta (1983) derived analytical equations for the average probability of allelic identity between tandemly arranged homologous genes under drift, mutation (μ per gene per generation), IGC (at rate c per gene per generation) and allelic crossover (at rate ρ per chromosome per generation). At equilibrium, the identity coefficient of the same block on different chromosomes (f), of different blocks on the same chromosome (c_1), and of different blocks on different chromosomes (c_2) are:

$$\hat{f} = \frac{2c\hat{c}_2 + \frac{1}{2N}}{2c + \frac{1}{2N} + 2\mu}, \quad (1)$$

$$\hat{c}_1 = \frac{\rho\hat{c}_2 + 2c}{\rho + 2c + 2\mu}, \quad (2)$$

and

$$\hat{c}_2 = \frac{c}{N}\left[\frac{4c + \frac{1}{2N} + 4\mu + \rho}{(2c+2\mu)\left(\frac{1}{2N}+2\mu\right)\left(4c+\frac{1}{2N}+2\mu\right) + \rho\left(\frac{c+\mu}{N} + 8c\mu + 4\mu + 4\mu^2\right)}\right]. \quad (3)$$

Model B (Innan 2002): Innan (2002) studied a two-locus two-allele IGC model to investigate the amounts of DNA variation and linkage disequilibrium (LD) in small multigene families. He presented exact solutions for the expectation values of variation within and between two loci. The expectation values of heterozygosity within each copy are identical between them and equal:

$$E(h_w) = 1 - 2\frac{\lambda}{\omega}, \quad (4)$$

where

$$\alpha = 2\theta + C,$$
$$\beta = 2 + 2\alpha + R,$$
$$\lambda = 4C^2 + \beta[2\theta C + 2\alpha(1+\theta)], \text{ and}$$
$$\omega = 8C^2 + 4\beta[\alpha(1+\alpha) - C^2].$$

The expectation of heterozygosity between the two loci is:

$$E(h_b) = 1 + \frac{1+\theta}{C} - \frac{2(1+\alpha)\lambda}{C\omega}. \quad (5)$$

It is worth noting that Ohta's expectations for the three types of identity coefficients are derived from transient equations (Ohta 1983) assuming small mutation, IGC, and recombination rates, whereas Innan uses a diffusion method and obtains exact solutions without this assumption (Innan 2002).

Model C (Innan 2003): A coalescent, infinite-site model of a small multigene family, also by Innan (2003), allowed him to obtain the expectations for average pairwise differences within loci $E(\pi_w)$ and between loci $E(\pi_b)$:

$$E(\pi_w) = \frac{2\Theta(2C+R+2)}{4C+R+2}, \quad (6)$$

$$E(\pi_b) = \frac{\Theta(4C^2+4C+2CR+R+2)}{C(4C+R+2)}, \quad (7)$$

where $\Theta = L\theta$, $\theta = 4N\mu$, $C = 4Nc = 4Ng\lambda$, and $R = 4Nr$.

Comparison between models

Figures S1 and S2 compare equations 1-7 with variation results from our simulations. In Figure S1, our simulations match the theoretical expectations for all the variation measures described above. Let us provide some more detail. We start by analyzing π_w^A and π_w^B, since their behavior is not entirely intuitive. Higher IGC rate implies a lower amount of variation found within each block. As can be seen in Figure S2, for any crossover rate and very high IGC rates, $\pi_w^B = \Theta$. Essentially, both blocks behave as if they were only one and maintain very high identity between them. As the IGC rate decreases, so does the proportion of segregating sites shared between blocks (Thornton 2007). However, the total amount of variation in each block increases due to the constant but low IGC activity until it reaches its maximum value of 2Θ. Higher crossover rates imply a higher independence between original and duplicated blocks, which in turn implies a higher effectiveness of IGC in transferring variants between duplicates. Therefore, the transition from Θ to 2Θ is shifted towards higher IGC rates as crossover rate increases. For $C < 0.01$ (or

$c < 2.5e{-6}$ and $N = 1000$, as in Figure S2), $\pi_w^B \approx 2\Theta$ irrespective of the crossover rate. Contrary to π_w^B, $\pi_w^A \rightarrow \Theta$ as $C \rightarrow 0$ which makes sense since without IGC, duplications evolve independently and the average pairwise differences within a block should equal Θ. In an infinite-site model, however, as our simulation results show, $\pi_w^B \rightarrow 2\Theta$ as $C \rightarrow 0$.

This apparent contradiction is solved once one considers that the time it takes to reach equilibrium increases drastically for very low IGC rates. Nagylaki (1984) described the rate of convergence to equilibrium and the asymptotic behavior of waiting times to reach equilibrium. This effect can be clearly seen in Figure S2, where the data from our simulations falls below the expected value in the infinite-site model. This is due precisely because for simulations for $C = 0.001$ have not yet reached equilibrium, even after 6 million generations. Further analysis and comparisons between the aforementioned equations can be found in File S5.

File S4

Validation on linkage disequilibrium

Innan (2002) also derived the expectation of LD between two loci:

$$E(D) = \frac{C}{\beta}\left(1 - \frac{2\lambda}{\omega}\right). \tag{8}$$

To generalize his theory to genes of length *L* (Innan 2003) he defined D_{sum} as the sum of LD at all *L* sites:

$$D_{sum} = \sum_{m=1}^{L} D_m, \tag{9}$$

where D_m is LD at site *m* (i.e. $D_m = \frac{n_{AA}n_{aa} - n_{Aa}n_{aA}}{n(n-1)}$, where n_{xy} represents the number of chromosomes with nucleotides *x* and *y* at original and duplicated genes, respectively). His expectation for D_{sum} for an infinite-site model (Innan 2003) is:

$$E(D_{sum}) = \frac{2\Theta C}{4C + R + 2}, \tag{10}$$

which is equivalent to E(*D*).

Figure S3 shows the results for D_{sum} from our simulations compared to E(D_{sum}). Our simulations show that E(D_{sum}) is not an accurate predictor for LD measures for high IGC rates when $R > 0$ since D_{sum} reaches a plateau before reaching Θ/2. This plateau is lower for higher crossover rates.

D_{sum} is a measure of LD between duplicate regions. To gain a deeper understanding of the pattern of LD not only between but within duplicates and in the whole region, we have calculated LD along the entire simulated region (see Methods).

File S5

Comparison between theoretical expectations of variation between duplicates

In this note, we analyze π_b^C, π_b^A and π_s^A. Recall that π_s^A corresponds to the average variation between duplicated blocks on the same chromosome while π_b^A is the average variation between different blocks on different chromosomes. This difference is reflected in their behavior for high IGC rates. As shown in Figure S4, $\pi_s^A \rightarrow 0$ while $\pi_b^A \rightarrow \Theta$ for high IGC rates. Of course, very high IGC rates will imply complete identity between duplicated blocks on the same chromosome while there is a minimum equilibrium divergence for blocks on different chromosomes in accordance with the neutral theory of molecular evolution.

For high IGC rates ($c >> \mu$) and very small crossover rates,

$$\pi_s^A(r \approx 0, c \gg \mu) = 1 - \hat{c}_1 \approx 1 - \frac{c}{c+\mu} = \frac{\mu}{c+\mu} \approx \frac{\mu}{c}. \quad (11)$$

For smaller conversion rates $\pi_b^A \approx \pi_s^A$, however, they diverge from π_b^C, as shown in Figure S4A. Interestingly, when $R = 0$, and contrary to what happens for high conversion rates, $\pi_s^A \neq \Theta/C$, but $\pi_b^C = \Theta/C$, since $C << 1$ (then, $C^2 << C$) and therefore,

$$\pi_b^C(R = 0, C \ll 1) = \frac{\Theta(4C^2 + 4C + 2)}{C(4C+2)} \approx \frac{\Theta(4C+2)}{C(4C+2)} = \frac{\Theta}{C} = \frac{\mu}{c}. \quad (12)$$

Additionally, we find that for $R = 0$, and for all values of C:

$$\frac{\Theta}{C} - \pi_b^C \approx \pi_s^A - \pi_b^A. \quad (13)$$

However, this is not the case for $R > 0$ as can be appreciated by comparing Figures S4A and S4B.

File S6

The effect on variability of allowing crossover in the whole simulated region

When crossovers are allowed along the entire simulated region (the WRC model, that contrasts with the more usual SCC model that allows crossover only in the single-copy region), variability descends to levels that are typical of crossover rates of one third of the ones implemented in the simulations of SCC. This can be explained if we recall that the effect of crossover between the original and the duplicated blocks is to allow IGC to transfer new mutations between blocks rather than moving the same variants from one block to its paralog over and over again. In other words, crossing over within the single-copy region breaks LD between the entire original and duplicated blocks. On the other hand, allowing crossover to occur outside the single-copy region breaks LD between some, but not all regions within the original and duplicated blocks.

Consider, for instance that a crossover junction falls 1 kbp from the 3' end of the duplicated block (L = 5 kbp). All regions right of the crossover junction will be effectively “cut off” from their paralogous region. However, all regions left of the block will remain linked with the original block. The effectiveness of crossover in this particular event will be reduced in four fifths since four fifths of the block will not be affected by crossover. If the crossover junction were to fall 1 kbp from the 5' end of the duplicated block, the effectiveness for crossover will only be reduced in one fifth. If we add up the reductions (of the effect of crossover) for all crossovers that fall within the duplicated block, we would expect an overall reduction of one half. The same is the case for crossovers that fall within the original block. If we consider that crossovers junctions fall homogeneously from 5' end of the original block to 3' end of the duplicated block, we will have no reduction from the one third of crossovers that fall in the single-copy block and one half reduction from crossovers that fall on either original or duplicated blocks, yielding and overall reduction of the effect of crossover of one third.

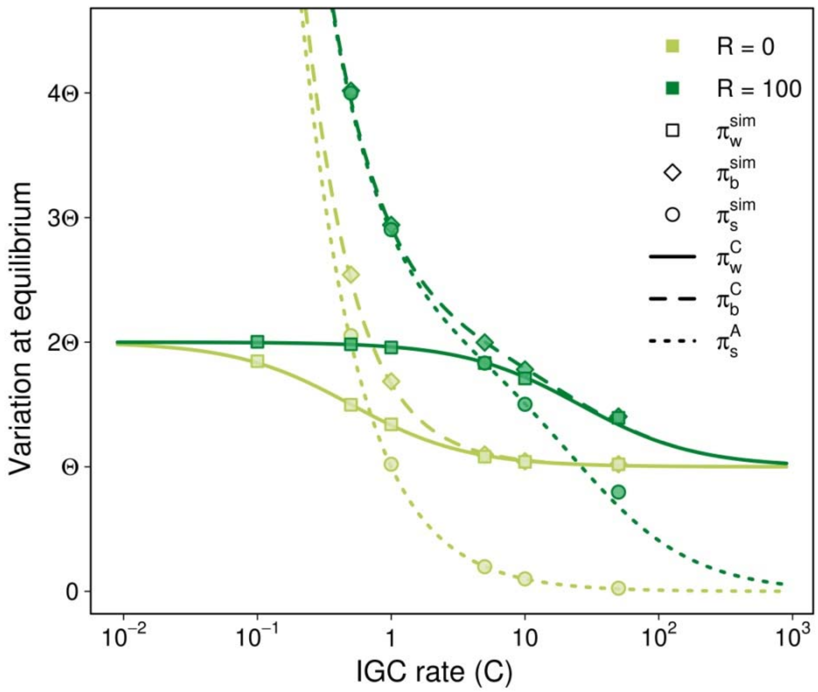

Figure S1 Comparison of main measures of variation. Theoretical expectations for variation within copies (π_w^C) (Innan 2003), variation between copies on different chromosomes (π_b^C) (Innan 2009), and variation between copies on the same chromosome (π_s^A) (Ohta 1983) are shown together with their corresponding values obtained by simulation (π_w^{sim}, π_b^{sim}, and π_s^{sim}). For high IGC rates, π_w^C and π_b^C converge to Θ, while π_s^A converges to 0 because high IGC homogenizes blocks on the same chromosome, but neutral variability found within populations is still present between different chromosomes. For low gene conversion values, π_s^A and π_b^C become identical and increase as IGC rate decreases, while $\pi_w^C \rightarrow 2\Theta$.

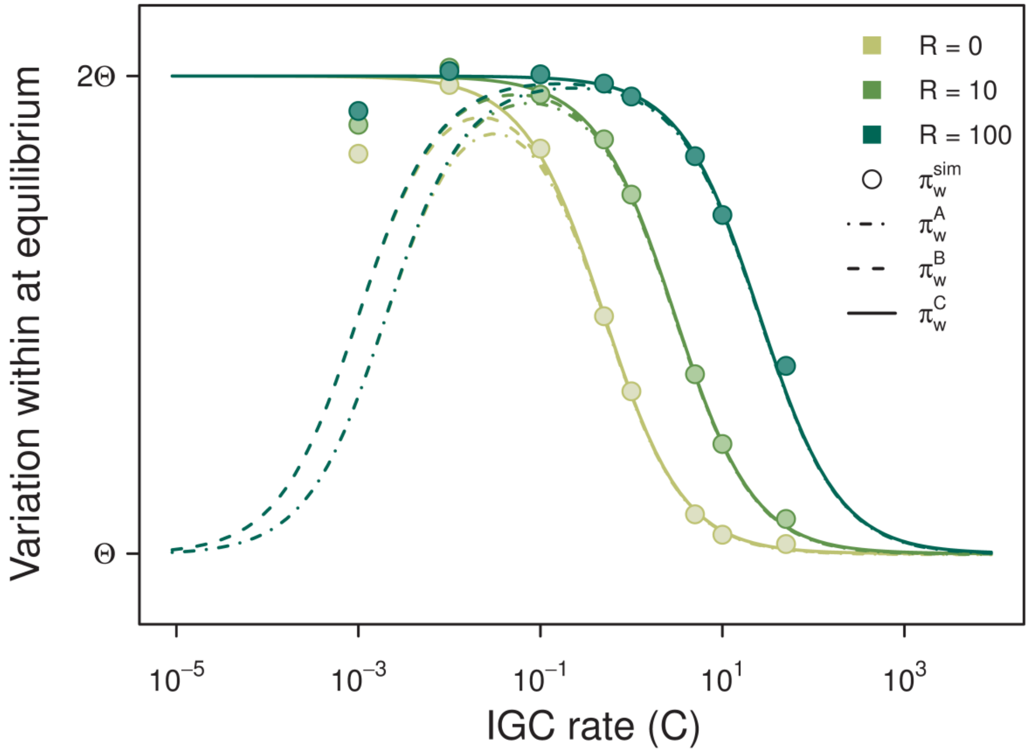

Figure S2 Comparison of variation within blocks between models. Theoretical lines are from Ohta 1982 (π_w^A), Innan 2002 (π_w^B) and Innan 2003 (π_w^C). Circles show results from simulations (π_w^{sim}) with increasingly higher running times in order to reach equilibrium for smaller IGC rates. Simulations were run for 100 thousand generations for *C* = 0.5, 1, 5, 10, 50; 3 million generations for *C* = 0.1, 0.01; and 6 million generations for *C* = 0.001. In the latter case, despite high running times, equilibrium has not yet been reached. Theoretical predictions do not take into account the increasingly high waiting times to reach equilibrium for low IGC rates and thus forward simulations might be very useful to have predictions in this regime.

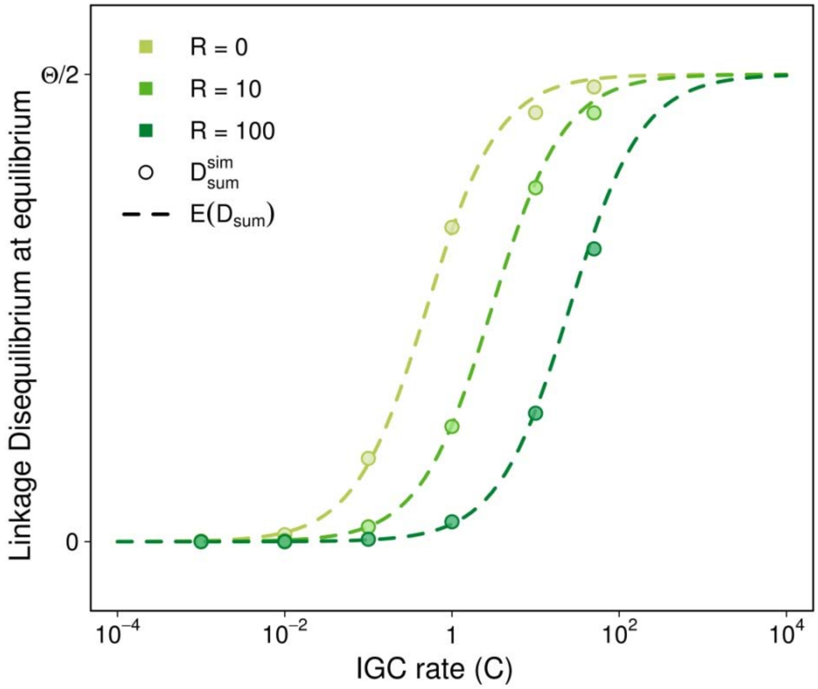

Figure S3 LD between duplicates. The theoretical expectations for LD between duplicates by Innan 2002 ($E(D_{sum})$) are shown together with the corresponding simulation values (D_{sum}^{sim}) for different IGC and crossover rates. LD between segmental duplications increases with IGC and decreases with crossover.

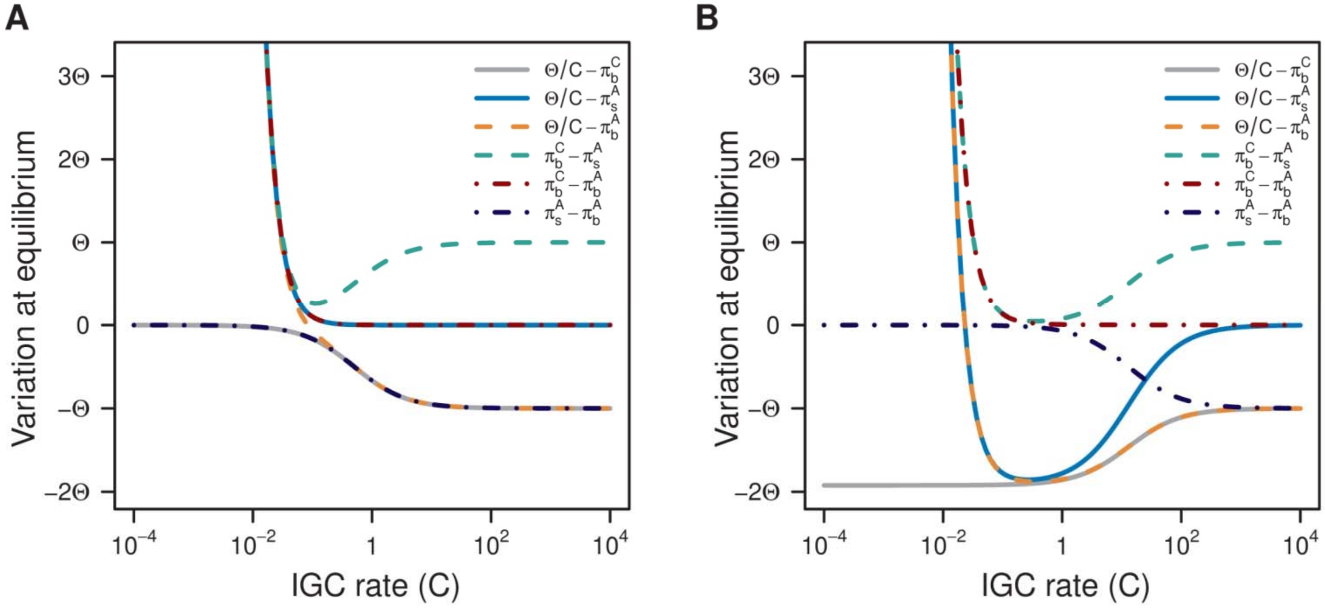

Figure S4 Comparison between different theoretical expectations of variation between duplicates. Figures show expectations for (**A**) R = 0 and (**B**) R = 50. Notice that $\Theta/C - \pi_b^C = \pi_s^A - \pi_b^A$ only for R = 0. For R > 0, $\Theta/C - \pi_b^C$ decreases with decreasing C as opposed to increasing constantly when R = 0. $\pi_s^A - \pi_b^A$, on the other hand, is independent of R.

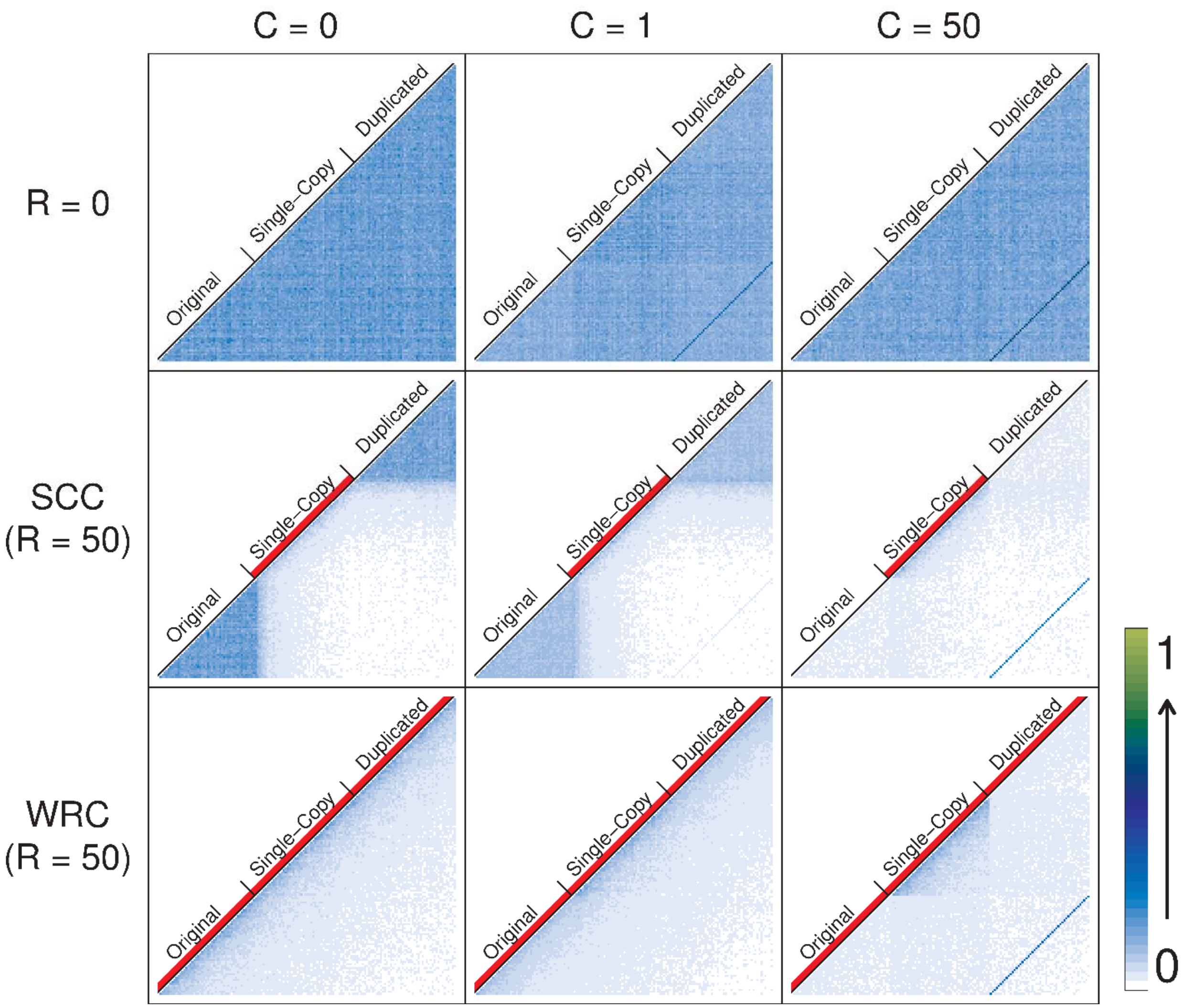

Figure S5 LD along the sequence under different crossover models. Here we use r^2 as a measure of LD. No significant differences can be observed when comparing with D' in Figure 4.

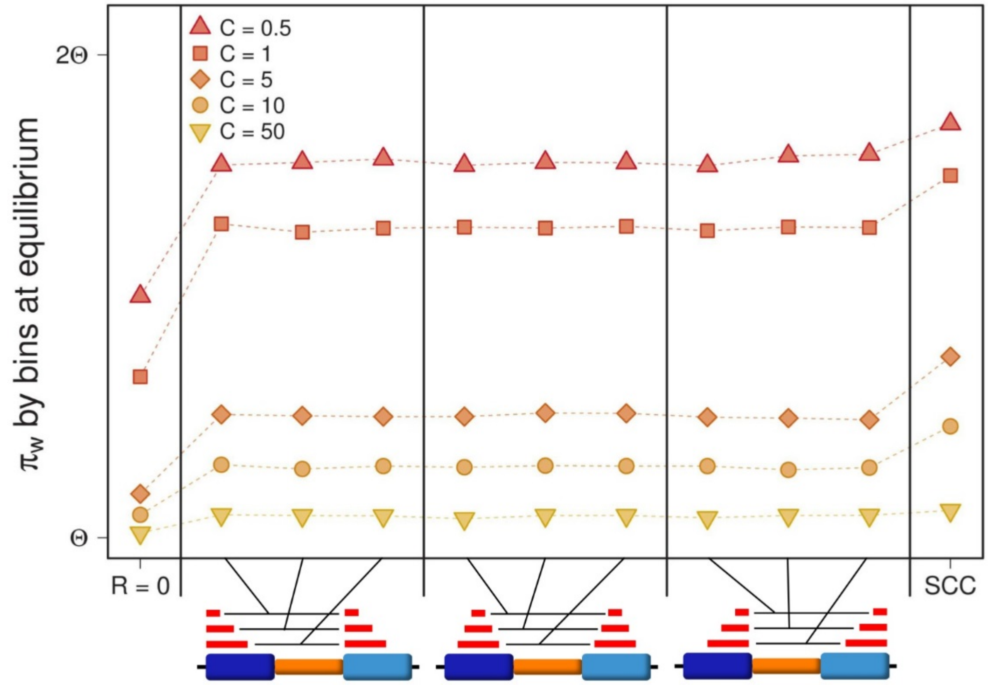

Figure S6 Variation within a duplicate block under different cases of HSC with two hotspots. In contrast to Figure 6 in which cases with a single hotspot are displayed, we show here scenarios with two hotspots present in equivalent locations of the two paralogous copies of the duplication. We observe that when there are two symmetrically located and equally active hotspots, there are no differences in levels of variation along the duplicates. Variation within a duplicate block for all two-hotspot HSC cases fall between the variation within blocks found when no crossover is acting (left) and the variation within blocks found under the SCC model (right), for the same value of *R* (R = 10).

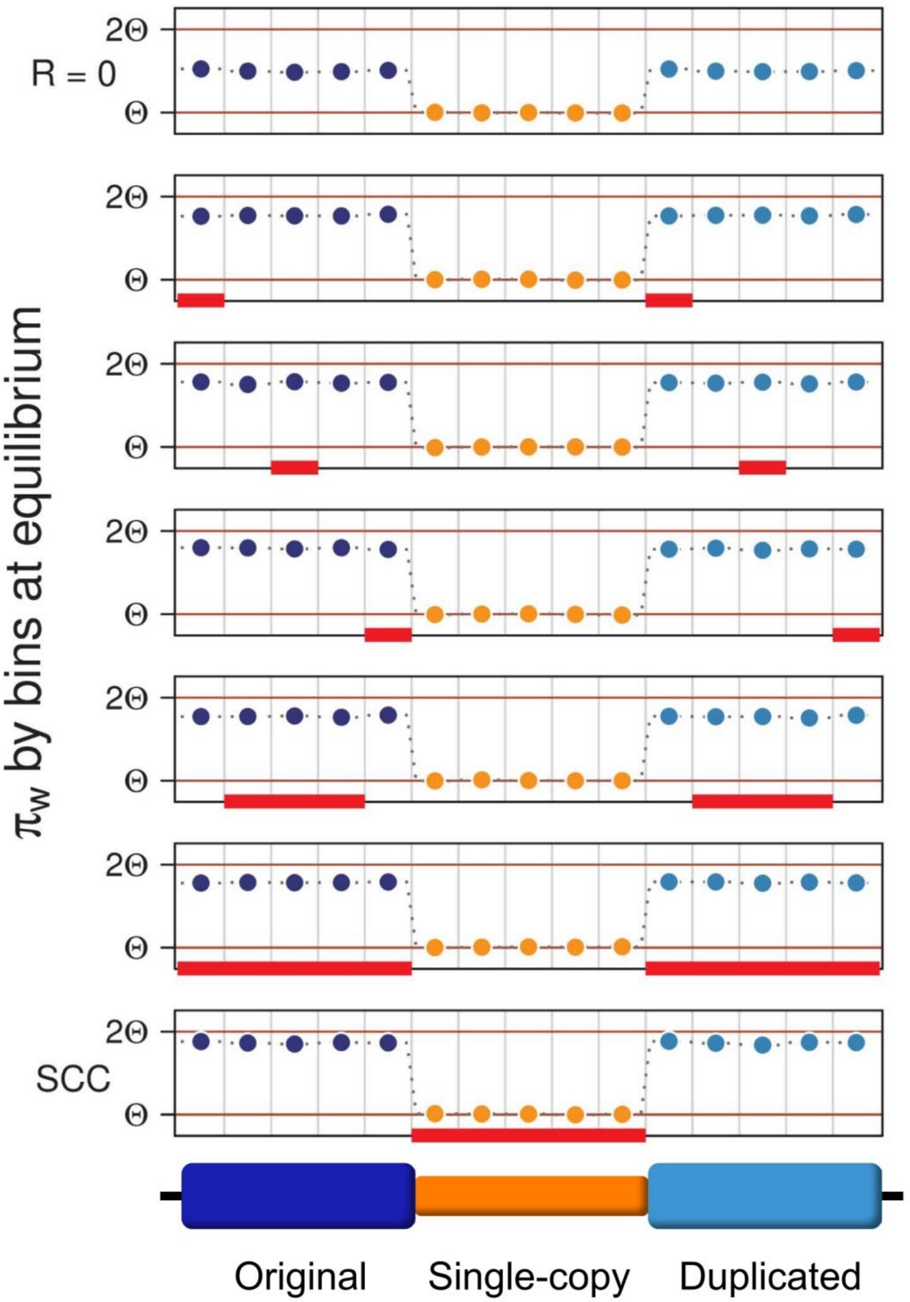

Figure S7 Distribution of variation along the simulated sequence under different two-hotspot HSC cases. The presence of two hotspots in paralogous regions on duplicate blocks homogenizes the amount of variation within all the bins of the duplicated blocks in all cases.

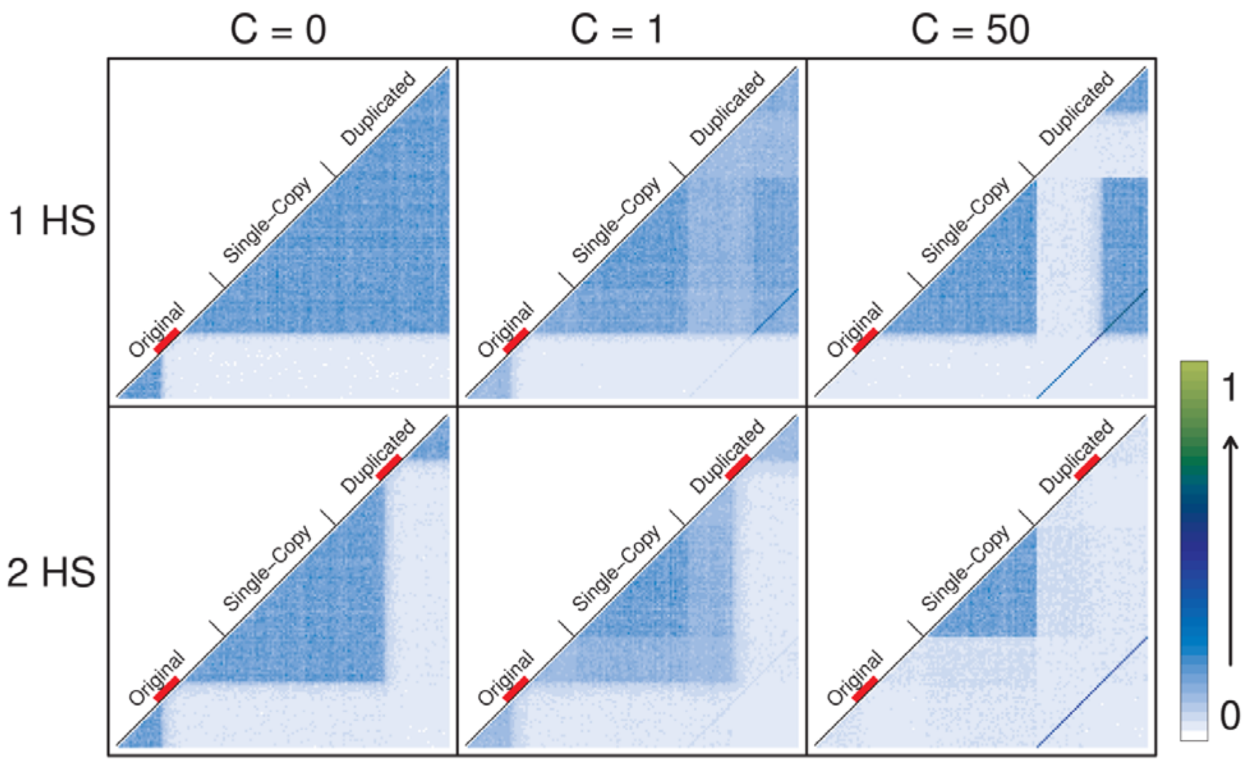

Figure S8 Comparison of LD along the sequence between models with one or two crossover hotspots. Here we use r^2 as a measure of LD. No significant differences can be observed when comparing with D' in Figure 8.

Additional References

Aleshin, A., and D. Zhi, 2010 Recombination-associated sequence homogenization of neighboring Alu elements: signature of nonallelic gene conversion. Mol. Biol. Evol. 27: 2300-2311.

Benovoy, D., and G. Drouin, 2009 Ectopic gene conversions in the human genome. Genomics 93: 27-32.

Bosch, E., M. E. Hurles, A. Navarro, and M. A. Jobling, 2004 Dynamics of a human interparalog gene conversion hotspot. Genome Res. 14: 835-844.

Casola, C., C. L. Ganote, and M. W. Hahn, 2010 Nonallelic gene conversion in the genus *Drosophila*. Genetics 185: 95-103.

Chen, J. M., D. N. Cooper, N. Chuzhanova, C. Férec, and G. P. Patrinos, 2007 Gene conversion: mechanisms, evolution and human disease. Nat. Rev. Genet. 8: 762-775.

Chen J. M., C. Férec, and D. N. Cooper, 2010 Gene conversion in human genetic disease. Genes 1: 550-563.

Collins A., J. Frezal, J. Teague, and N. E. Morton, 1996 A metric map of humans: 23,500 loci in 850 bands. Proc. Natl. Acad. Sci. USA 93: 14771-14775.

Ezawa, K., S. OOta, and N. Saitou, 2006 Proceedings of the SMBE Tri-National Young Investigators' Workshop 2005. Genome-wide search of gene conversions in duplicated genes of mouse and rat. Mol. Biol. Evol. 23: 927-940.

Goldman, A. S. H., and M. Lichten, 1996 The efficiency of meiotic recombination between dispersed sequences in *Saccharomyces cerevisiae* depends upon their chromosomal location. Genetics 144: 43-55.

Innan, H., 2002 A method for estimating the mutation, gene conversion and recombination parameters in small multigene families. Genetics 161: 865-872.

Innan, H., 2003 The coalescent and infinite-site model of a small multigene family. Genetics 163: 803-810.

Jeffreys, A. J., and C. A. May, 2004 Intense and highly localized gene conversion activity in human meiotic crossover hot spots. Nat. Genet. 36: 151-156.

Lichten, M., H. Rhona, and J. E. Haber, 1987 Meiotic gene conversion and crossing over between dispersed homologous sequences occurs frequently in *Saccharomyces cerevisiae*. Genetics 115: 233-246.

Lichten, M., and J. E. Haber, 1989 Position effects in ectopic and allelic mitotic reacombination in *Saccaromyces cerevisiae*. Genetics 123: 261-268.

Liskay, R. M., A. Letsou, and J. L. Stachelek, 1987 Homology requirement for efficient gene conversion between duplicated chromosomal sequences in mammalian cells. Genetics 115: 161-167.

Lukacsovich, T., and A. S. Waldman, 1999 Suppression of intrachromosomal gene conversion in mammalian cells by small degrees of sequence divergence. Genetics 151: 1559-1568.

Mansai, S. P., and H. Innan, 2010 The power of the methods for detecting interlocus gene conversion. Genetics 184: 517-527.

Mansai, S. P., T. Kado, and H. Innan, 2011 The rate and tract length of gene conversion between duplicated genes. Genes 2: 313-331.

McGrath, C. L., C. Casola, and M. W. Hahn, 2009 Minimal effect of ectopic gene conversion among recent duplicates in four mammalian genomes. Genetics 182: 615-622.

McVean, G. A. T., S. R. Myers, S. Hunt, P. Deloukas, D. R. Bentley *et al*., 2004 The fine-scale structure of recombination rate variation in the human genome. Science 304: 581-584.

Nagylaki, T., 1984 The evolution of multigene families under intrachromosomal gene conversion. Genetics 106: 529-548.

Ohta, T., 1983 On the evolution of multigene families. Theor. Popul. Biol. 23: 216-240.

Petes, T. D., and C. W. Hill, 1988 Recombination between repeated genes in mictroorganisms. Annu. Rev. Genet. 22: 147-168.

Reiter, L. T., P. J. Hastings, E. Nelis, P. De Jonghe, C. Van Broeckhoven *et al*., 1998 Human meiotic recombination products revealed by sequencing a hotspot for homologous strand exchange in multiple HNPP deletion patients. Am. J. Hum. Genet. 62: 1023-1033.

Sawyer, S., 1989 Statistical tests for detecting gene conversion. Mol. Biol. Evol. 6: 526-538.

Semple, C., and K. H. Wolfe, 1999 Gene duplication and gene conversion in the *Caenorhabditis elegans* genome. J. Mol. Evol. 48: 555-564.

Shen, P., and H. V. Huang, 1986 Homologous recombination in *Escherichia coli*: dependence on substrate length and homology. Genetics 112: 441-457.

Sudmant, P. H., J. O. Kitzman, F. Antonacci, C. Alkan, M. Malig *et al*., 2010 Diversity of human copy number variation and multicopy genes. Science 330: 641-646.

Thornton, K. R., 2007 The neutral coalescent process for recent gene duplications and copy-number variants. Genetics 177: 987–1000.

Waldman A. S., and R. M. Liskay, 1988 Dependence of intrachromosomal recombination in mammalian cells on uninterrupted homology. Mol. Cell. Biol. 8: 5350-5357.

Walsh, J. B., 1987 Sequence-dependent gene conversion: can duplicated genes diverge fast enough to escape conversion? Genetics 117: 543-557.
