# Supplementary material for: Interplay of Interlocus Gene Conversion and Crossover in Segmental Duplications Under a Neutral Scenario
Source: G3 (Bethesda). 2014 Jun 6;4(8):1479–89. doi: 10.1534/g3.114.012435 (PMC4132178; doi:10.1534/g3.114.012435)
Supplement: Supporting Information [file supp_g3.114.012435_FileS1.pdf]

File S1

Parameter Values

The range of parameters we have explored is based primarily on previous coalescent simulations by Innan (2003) and Thornton (2007). The parameter values explored here are compared to theirs in Table 1 in the main text. Here we will also consider different crossover models.

IGC rates

The rate of IGC has been shown to depend on a series of factors, reviewed by Chen *et al*. (2007) and discussed in detail by Mansai *et al*. (2011), who contrast empirical and evolutionary approaches to measure IGC rates; by Benvoy and Drouin (2009), who analyze IGC between human protein coding genes; by McGrath *et al.* (2009), who do so for human, macaque, mouse and rat; and Casola *et al.* (2010) in the genus *Drosophila*. Here we will briefly mention these factors in order to justify our choice of parameters, and also to clarify that IGC models like the one we implement here are still far from being close to reality, in part because of a lack of consistency between different studies on IGC rates. In our simulations, we test IGC rates of C from 0.001 to 100 in populations of N = 1000. Therefore, IGC rates per site per generation (c) range from $2.5\text{x}10^{-7}$ to $2.5\text{x}10^{-2}$.

Organism: Genome architecture generates differences in IGC rate between organisms, with estimates ranging, for instance, from $\sim 10^{-10}$ to $\sim 10^{-3}$ in *S. cerevisiae* (cited in Mansai *et al.* 2011) to $\sim 10^{-4}$ to $\sim 10^{-3}$ in humans (Chen *et al.* 2007).

Meiosis vs. mitosis: IGC rate has been reported to be from ~15 to ~300 times higher in meiosis than in mitosis in yeast (cited in Mansai *et al.* 2011).

Intrachromosomal vs. interchromosomal: In humans, intrachromosomal IGC events are ~5 times more frequent than their interchromosomal equivalent (Benovoy and Drouin 2009). A similar relationship has been found in mammals (McGrath *et al*. 2009), yeast (Petes and Hill 1988) and *C. elegans* (Semple and Wolfe 1999). Here we exclusively simulate meiotic intrachromosomal IGC events.

Distance between duplicates: The rate of IGC seems to be negatively correlated with distance between the duplicated regions involved in intrachromosomal IGC events (Lichten and Haber 1989; Semple and Wolfe 1999; Benovoy and Drouin 2009; Aleshin and Zhi 2010; Casola *et al.* 2010). This could in fact be a consequence of neighboring paralogs being more recent and therefore less divergent on average, implying higher IGC rates (McGrath *et al.* 2009). In humans, the median distance between intrachromosomal converted genes is $7.8\text{x}10^4 \pm 2.88\text{x}10^7$ bp and more than 50% of conversions occur between genes that are less than 10^4 bp apart (Benovoy and Drouin 2009). For more details, see File S2.

Orientation: Although there is an excess of IGC events between genes with the same orientation (Semple and Wolfe 1999; Benovoy and Drouin 2009; Aleshin and Zhi 2010), it might be the consequence of an excess of close-by duplicated genes (less than 50 kb from each other) in a head-to-tail orientation (McGrath *et al.* 2009).

Sequence similarity: IGC rates seem to be positively correlated with DNA sequence similarity between converted pairs of genes in unconverted regions (Semple and Wolfe 1999) with most conversions occurring for similarity levels above 95% (Chen *et al*. 2007; Sudmant *et al*. 2010). The strength of this correlation might be biased because the most commonly used software to detect conversions (GENECONV by Sawyer *et al.* 1989) lacks power when divergence between duplicates is very low (McGrath e*t al*. 2009). The minimal degree of identity necessary for IGC to occur or *minimal efficient sequence homology* (MESH) is ~92% (Chen *et al.* 2010). Walsh (1987) examined models under which IGC frequencies decreased due to sequence divergence between duplicates. Considering a model where IGC rates decrease linearly with sequence divergence and exclusively through accumulation of point mutations, Walsh states that if $2\mu/c << 0.1$ genes never escape IGC. Setting $\theta = 0.001$ and considering $2\theta/C = 0.01$ as our threshold, this would imply that IGC rates $C > 0.2$ would ensure stable concerted evolution. In this work, we consider IGC to act without any restriction on sequence similarity but concentrate our analyses on IGC rates $C > 0.2.$

Sequence identity: Additional to the overall level of divergence between duplicated regions, there appears to be a *minimal efficient processing segment* (MEPS) (Shen and Huang 1986); for there to be effective gene conversion, a segment of 100% identity needs to be adjacent to the gene conversion tract. Measurements of MEPS in mammalian cells fall around 200 bp: between 200 and 295 bp (Liskay *et al*. 1987) and between 134 and 232 bp (Waldman and Liskay 1988). MEPS for humans has been estimated to lie between 337 and 456 bp (Reiter *et al*. 1998), although they can be as short as 40 bp for *Alu* repeats (Aleshin and Zhi 2010). In this study, we allow IGC to act irrespective of the existence of a MEPS. The inclusion of MESH and MEPS in future studies would be of relevance, particularly in the case of low IGC rates.

Local crossover rates: We know that IGC is associated with crossover of flanking sequences (Lichten *et al.* 1987), and it would therefore make sense for local IGC rate to be dependent on local crossover rates. Significant (Benovoy and Drouin 2009) and non-significant (McGrath *et al*. 2009) correlations between frequency of IGC events and local rates of recombination have been reported.

Gene conversion tract lengths

Although most available estimates on gene conversion tract lengths are from intralocus gene conversion, the molecular mechanism involved in both intralocus and interlocus (IGC) gene conversion is generally considered to be the same (Jeffreys and May 2004). Estimates for average tract lengths are extracted from measurements of minimum and maximum values and range from 55-290 bp for the average gene conversion tract length in human hotspot DNA3 (Jeffreys and May 2004), to 210 bp in human genes shared with macaque, mouse and rat (McGrath *et al*. 2009), to 31 bp (assuming a gene conversion event to have occurred if at least two variant sites have been converted) for two human endogenous retroviral (HERV) sequences located on chromosome Y (Bosch *et al.* 2004). For human protein coding genes, a maximum of 10 bp and a maximum of 6011 bp have been detected (Benovoy and Drouin 2009), but the latter might be the case of several overlapping gene conversions, since according to Chen *et al*. (2007), single gene conversion events rarely exceed 1 kb in humans.

There is evidence suggesting that gene conversion tract lengths depend on the level of sequence divergence between interacting sequences (Lukacsovish and Waldman 1999). Considering the maximum sequence similarity between the 100 nucleotides upstream and downstream of the gene conversion event, Benovoy and Drouin (2009) show that gene conversion events shorter than 1 kb in length can occur with flanking regions with sequence similarity as low as 23.8%, whereas larger than 1 kb gene conversion events only occur is sequence similarity is above 89%. This finding might imply a cutoff in the gene conversion machinery if sequence similarity happens to fall (Benovoy and Drouin 2009), although, to the best of our knowledge, this has not been formally tested.

Average IGC tract length estimates for other organisms such as yeast and rodents are also close to 100 bp (Mansai *et al.* 2011). In simulations, common average conversion tract lengths used range from 100 to 1000 bp (Thornton 2007; Mansai and Innan 2010). Throughout this work the average IGC tract length is $\lambda = 100$ bp.

Recombination

In 1996, Collins *et al*. established a sex-averaged figure of 0.88 Mb equal to 1 cM in humans (Collins *et al.* 1996). Since then, an approximation of 1 cM/Mb (0.01 crossovers per generation per Mb) has been adopted as a consensus figure that results in a per nucleotide per generation homologous recombination rate of 1×10^{-8}. Since they are genome-wide averages, these figures include crossover activity in hotspots. If we were to consider that there are no hotspots within the region we simulate, it would be better to consider the background crossover rate, estimated to be of 0.1 cM/Mb that is, 10^{-9} recombination events per nucleotide per generation (McVean *et al*. 2004).

We test crossover rates of R = 0, 1, 10, 50, and 100, in populations of N = 1000. Therefore, crossover rates per chromosome per generation (r) vary from 2.5×10^{-4} to 2.5×10^{-2} (evidently, excluding the case of r = 0). In the case of SCC (and L = 5000), these correspond to per-site crossover rates from 5×10^{-8} to 5×10^{-6}; for WRC, from 1.7×10^{-8} to 1.7×10^{-6}, and for HSC with 1 kb hotspots, from 2.5×10^{-7} to 2.5×10^{-5}.
In this paper, we ignore homologous recombination resolution by intralocus gene conversion, we exclusively simulate crossover (meaning exchange of tails) to one side of the crossover junction.
