# Supplementary material for: Interplay of Interlocus Gene Conversion and Crossover in Segmental Duplications Under a Neutral Scenario
Source: G3 (Bethesda). 2014 Jun 6;4(8):1479–89. doi: 10.1534/g3.114.012435 (PMC4132178; doi:10.1534/g3.114.012435)
Supplement: Supporting Information [file supp_g3.114.012435_FileS2.pdf]

File S2

Dependence of IGC rate on distance between duplicates

There is evidence from many species that the frequency of intrachromosomal IGC events increases as the distance between the implicated genes decreases [humans (Benovoy and Drouin 2009; McGrath *et al*. 2009), mouse (Ezawa *et al*. 2006; McGrath *et al*. 2009), *Drosophila* (Casola *et al.* 2010), yeast (Goldman and Lichten 1996) and *C. elegans* (Semple and Wolfe 1999)]. However, this excess of IGC activity is attributed to different causes by different authors. Benovoy and Drouin (2009) attribute it to the fact that most duplicated functional genes lie in close vicinity to each other. They argue that the majority of gene family members are within 10 kbp of each other whereas most unrelated genes lay further away (Benovoy and Drouin 2009). Interestingly, McGrath and collaborators argue that the inverse proportionality between physical distance and frequency of IGC might just be a consequence of less divergence among closely placed duplications and not a direct consequence of the physical distance between them. Since many duplications arise through NAHR, neighboring duplicates are likely to have a more recent origin and thus be less divergent between them. Discriminating between these confounding factors is complicated since an IGC event directly affects divergence, and IGCs between highly similar sequences are difficult to detect (Mansai and Innan, 2010). In mammals, results show that once divergence between duplicates is accounted for, there is no significant relationship between physical distance and IGC rates (McGrath *et al*., 2009).

Regardless of its underlying cause, there is a negative correlation between IGC rates and physical distance between duplicates. It is clear that increasing the distance between two points along a chromosome will tend to increase the chance for there to be a crossover between them. Therefore, crossover and IGC rates have, respectively, a positive and negative correlation with physical distance between duplicates. Since equilibrium diversity within each block is also positively correlated with crossover rates between blocks, but negatively correlated with IGC rates, we can expect, in principle, a positive correlation between diversity in duplicated blocks and the distance between them. Closely located duplicates will be expected to have higher identity and lower diversity than duplicates further away from each other.
