# Supplementary material for: Interplay of Interlocus Gene Conversion and Crossover in Segmental Duplications Under a Neutral Scenario
Source: G3 (Bethesda). 2014 Jun 6;4(8):1479–89. doi: 10.1534/g3.114.012435 (PMC4132178; doi:10.1534/g3.114.012435)
Supplement: Supporting Information [file supp_g3.114.012435_FileS3.pdf]

File S3

Validation on variation

Increased variation due to the effect of IGC has been extensively studied and modeled for small and large multigene families. We have selected three different models (Ohta 1983; Innan 2002, 2003) in order to validate the results of our simulations.

Model A (Ohta 1983): Ohta (1983) derived analytical equations for the average probability of allelic identity between tandemly arranged homologous genes under drift, mutation (μ per gene per generation), IGC (at rate *c* per gene per generation) and allelic crossover (at rate ρ per chromosome per generation). At equilibrium, the identity coefficient of the same block on different chromosomes (*f*), of different blocks on the same chromosome (c_1), and of different blocks on different chromosomes (c_2) are:

$$\hat{f} = \frac{2c\hat{c}_2 + \frac{1}{2N}}{2c + \frac{1}{2N} + 2\mu}, \quad (1)$$

$$\hat{c}_1 = \frac{\rho\hat{c}_2 + 2c}{\rho + 2c + 2\mu}, \quad (2)$$

and

$$\hat{c}_2 = \frac{c}{N}\left[\frac{4c + \frac{1}{2N} + 4\mu + \rho}{(2c+2\mu)\left(\frac{1}{2N}+2\mu\right)\left(4c+\frac{1}{2N}+2\mu\right) + \rho\left(\frac{c+\mu}{N} + 8c\mu + 4\mu + 4\mu^2\right)}\right]. \quad (3)$$

Model B (Innan 2002): Innan (2002) studied a two-locus two-allele IGC model to investigate the amounts of DNA variation and linkage disequilibrium (LD) in small multigene families. He presented exact solutions for the expectation values of variation within and between two loci. The expectation values of heterozygosity within each copy are identical between them and equal:

$$E(h_w) = 1 - 2\frac{\lambda}{\omega}, \quad (4)$$

where

$$\alpha = 2\theta + C,$$
$$\beta = 2 + 2\alpha + R,$$
$$\lambda = 4C^2 + \beta[2\theta C + 2\alpha(1+\theta)], \text{ and}$$
$$\omega = 8C^2 + 4\beta[\alpha(1+\alpha) - C^2].$$

The expectation of heterozygosity between the two loci is:

$$E(h_b) = 1 + \frac{1+\theta}{C} - \frac{2(1+\alpha)\lambda}{C\omega}. \quad (5)$$

It is worth noting that Ohta's expectations for the three types of identity coefficients are derived from transient equations (Ohta 1983) assuming small mutation, IGC, and recombination rates, whereas Innan uses a diffusion method and obtains exact solutions without this assumption (Innan 2002).

Model C (Innan 2003): A coalescent, infinite-site model of a small multigene family, also by Innan (2003), allowed him to obtain the expectations for average pairwise differences within loci $E(\pi_w)$ and between loci $E(\pi_b)$:

$$E(\pi_w) = \frac{2\Theta(2C+R+2)}{4C+R+2}, \quad (6)$$

$$E(\pi_b) = \frac{\Theta(4C^2+4C+2CR+R+2)}{C(4C+R+2)}, \quad (7)$$

where $\Theta = L\theta$, $\theta = 4N\mu$, $C = 4Nc = 4Ng\lambda$, and $R = 4Nr$.

Comparison between models

Figures S1 and S2 compare equations 1-7 with variation results from our simulations. In Figure S1, our simulations match the theoretical expectations for all the variation measures described above. Let us provide some more detail. We start by analyzing π_w^A and π_w^B, since their behavior is not entirely intuitive. Higher IGC rate implies a lower amount of variation found within each block. As can be seen in Figure S2, for any crossover rate and very high IGC rates, $\pi_w^B = \Theta$. Essentially, both blocks behave as if they were only one and maintain very high identity between them. As the IGC rate decreases, so does the proportion of segregating sites shared between blocks (Thornton 2007). However, the total amount of variation in each block increases due to the constant but low IGC activity until it reaches its maximum value of 2Θ. Higher crossover rates imply a higher independence between original and duplicated blocks, which in turn implies a higher effectiveness of IGC in transferring variants between duplicates. Therefore, the transition from Θ to 2Θ is shifted towards higher IGC rates as crossover rate increases. For $C < 0.01$ (or

c < 2.5e-6 and *N* = 1000, as in Figure S2), $\pi_w^B \approx 2\Theta$ irrespective of the crossover rate. Contrary to π_w^B, $\pi_w^A \rightarrow \Theta$ as $C \rightarrow 0$ which makes sense since without IGC, duplications evolve independently and the average pairwise differences within a block should equal Θ. In an infinite-site model, however, as our simulation results show, $\pi_w^B \rightarrow 2\Theta$ as $C \rightarrow 0$.

This apparent contradiction is solved once one considers that the time it takes to reach equilibrium increases drastically for very low IGC rates. Nagylaki (1984) described the rate of convergence to equilibrium and the asymptotic behavior of waiting times to reach equilibrium. This effect can be clearly seen in Figure S2, where the data from our simulations falls below the expected value in the infinite-site model. This is due precisely because for simulations for *C* = 0.001 have not yet reached equilibrium, even after 6 million generations. Further analysis and comparisons between the aforementioned equations can be found in File S5.
