# Supplementary material for: Interplay of Interlocus Gene Conversion and Crossover in Segmental Duplications Under a Neutral Scenario
Source: G3 (Bethesda). 2014 Jun 6;4(8):1479–89. doi: 10.1534/g3.114.012435 (PMC4132178; doi:10.1534/g3.114.012435)
Supplement: Supporting Information [file supp_g3.114.012435_FileS4.pdf]

File S4

Validation on linkage disequilibrium

Innan (2002) also derived the expectation of LD between two loci:

$$E(D) = \frac{C}{\beta}\left(1 - \frac{2\lambda}{\omega}\right). \quad (8)$$

To generalize his theory to genes of length L (Innan 2003) he defined D_{sum} as the sum of LD at all L sites:

$$D_{sum} = \sum_{m=1}^{L} D_m, \quad (9)$$

where D_m is LD at site m (i.e. $D_m = \frac{n_{AA}n_{aa} - n_{Aa}n_{aA}}{n(n-1)}$, where n_{xy} represents the number of chromosomes with nucleotides x and y at original and duplicated genes, respectively). His expectation for D_{sum} for an infinite-site model (Innan 2003) is:

$$E(D_{sum}) = \frac{2\Theta C}{4C + R + 2}, \quad (10)$$

which is equivalent to E(D).

Figure S3 shows the results for D_{sum} from our simulations compared to E(D_{sum}). Our simulations show that E(D_{sum}) is not an accurate predictor for LD measures for high IGC rates when $R > 0$ since D_{sum} reaches a plateau before reaching Θ/2. This plateau is lower for higher crossover rates.

D_{sum} is a measure of LD between duplicate regions. To gain a deeper understanding of the pattern of LD not only between but within duplicates and in the whole region, we have calculated LD along the entire simulated region (see Methods).
