# Supplementary material for: Interplay of Interlocus Gene Conversion and Crossover in Segmental Duplications Under a Neutral Scenario
Source: G3 (Bethesda). 2014 Jun 6;4(8):1479–89. doi: 10.1534/g3.114.012435 (PMC4132178; doi:10.1534/g3.114.012435)
Supplement: Supporting Information [file supp_g3.114.012435_FileS5.pdf]

File S5

Comparison between theoretical expectations of variation between duplicates

In this note, we analyze π_b^C, π_b^A and π_s^A. Recall that π_s^A corresponds to the average variation between duplicated blocks on the same chromosome while π_b^A is the average variation between different blocks on different chromosomes. This difference is reflected in their behavior for high IGC rates. As shown in Figure S4, $\pi_s^A \rightarrow 0$ while $\pi_b^A \rightarrow \Theta$ for high IGC rates. Of course, very high IGC rates will imply complete identity between duplicated blocks on the same chromosome while there is a minimum equilibrium divergence for blocks on different chromosomes in accordance with the neutral theory of molecular evolution.

For high IGC rates ($c >> \mu$) and very small crossover rates,

$$\pi_s^A(r \approx 0, c \gg \mu) = 1 - \hat{c}_1 \approx 1 - \frac{c}{c+\mu} = \frac{\mu}{c+\mu} \approx \frac{\mu}{c}. \tag{11}$$

For smaller conversion rates $\pi_b^A \approx \pi_s^A$, however, they diverge from π_b^C, as shown in Figure S4A. Interestingly, when $R = 0$, and contrary to what happens for high conversion rates, $\pi_s^A \neq \Theta/C$, but $\pi_b^C = \Theta/C$, since $C << 1$ (then, $C^2 << C$) and therefore,

$$\pi_b^C(R = 0, C \ll 1) = \frac{\Theta(4C^2 + 4C + 2)}{C(4C+2)} \approx \frac{\Theta(4C+2)}{C(4C+2)} = \frac{\Theta}{C} = \frac{\mu}{c}. \tag{12}$$

Additionally, we find that for $R = 0$, and for all values of C:

$$\frac{\Theta}{C} - \pi_b^C \approx \pi_s^A - \pi_b^A. \tag{13}$$

However, this is not the case for $R > 0$ as can be appreciated by comparing Figures S4A and S4B.
