# Supplementary material for: Interplay of Interlocus Gene Conversion and Crossover in Segmental Duplications Under a Neutral Scenario
Source: G3 (Bethesda). 2014 Jun 6;4(8):1479–89. doi: 10.1534/g3.114.012435 (PMC4132178; doi:10.1534/g3.114.012435)
Supplement: Supporting Information [file supp_g3.114.012435_FileS6.pdf]

File S6

The effect on variability of allowing crossover in the whole simulated region

When crossovers are allowed along the entire simulated region (the WRC model, that contrasts with the more usual SCC model that allows crossover only in the single-copy region), variability descends to levels that are typical of crossover rates of one third of the ones implemented in the simulations of SCC. This can be explained if we recall that the effect of crossover between the original and the duplicated blocks is to allow IGC to transfer new mutations between blocks rather than moving the same variants from one block to its paralog over and over again. In other words, crossing over within the single-copy region breaks LD between the entire original and duplicated blocks. On the other hand, allowing crossover to occur outside the single-copy region breaks LD between some, but not all regions within the original and duplicated blocks.

Consider, for instance that a crossover junction falls 1 kbp from the 3' end of the duplicated block (L = 5 kbp). All regions right of the crossover junction will be effectively “cut off” from their paralogous region. However, all regions left of the block will remain linked with the original block. The effectiveness of crossover in this particular event will be reduced in four fifths since four fifths of the block will not be affected by crossover. If the crossover junction were to fall 1 kbp from the 5' end of the duplicated block, the effectiveness for crossover will only be reduced in one fifth. If we add up the reductions (of the effect of crossover) for all crossovers that fall within the duplicated block, we would expect an overall reduction of one half. The same is the case for crossovers that fall within the original block. If we consider that crossovers junctions fall homogeneously from 5' end of the original block to 3' end of the duplicated block, we will have no reduction from the one third of crossovers that fall in the single-copy block and one half reduction from crossovers that fall on either original or duplicated blocks, yielding and overall reduction of the effect of crossover of one third.
