# Supplementary material for: Interplay of Interlocus Gene Conversion and Crossover in Segmental Duplications Under a Neutral Scenario
Source: G3 (Bethesda). 2014 Jun 6;4(8):1479–89. doi: 10.1534/g3.114.012435 (PMC4132178; doi:10.1534/g3.114.012435)
Supplement: Supporting Information [file supp_g3.114.012435_FigureS1.pdf]

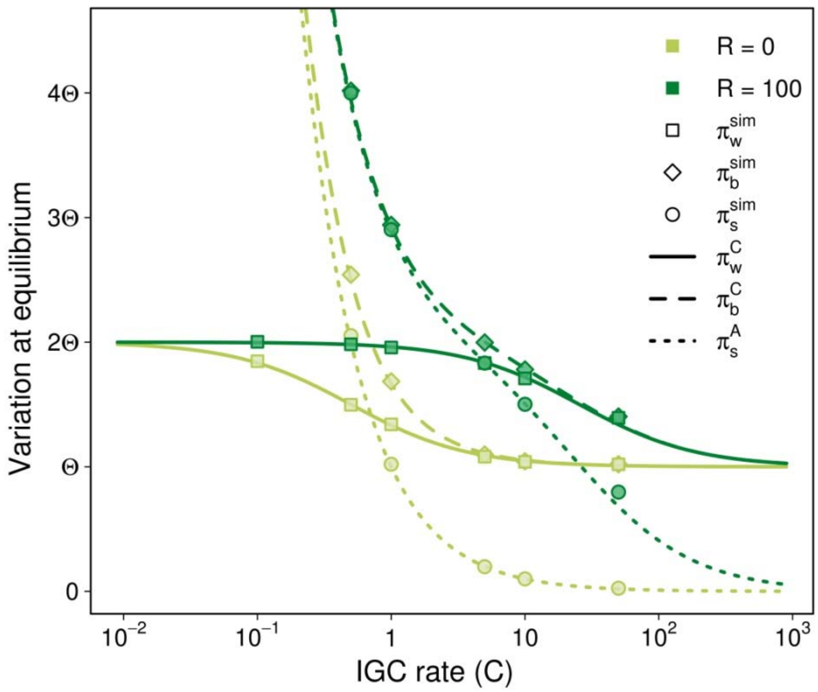

Figure S1 Comparison of main measures of variation. Theoretical expectations for variation within copies (π_w^C) (Innan 2003), variation between copies on different chromosomes (π_b^C) (Innan 2009), and variation between copies on the same chromosome (π_s^A) (Ohta 1983) are shown together with their corresponding values obtained by simulation (π_w^{sim}, π_b^{sim}, and π_s^{sim}). For high IGC rates, π_w^C and π_b^C converge to Θ, while π_s^A converges to 0 because high IGC homogenizes blocks on the same chromosome, but neutral variability found within populations is still present between different chromosomes. For low gene conversion values, π_s^A and π_b^C become identical and increase as IGC rate decreases, while $\pi_w^C \rightarrow 2\Theta$.
