# Supplementary material for: Interplay of Interlocus Gene Conversion and Crossover in Segmental Duplications Under a Neutral Scenario
Source: G3 (Bethesda). 2014 Jun 6;4(8):1479–89. doi: 10.1534/g3.114.012435 (PMC4132178; doi:10.1534/g3.114.012435)
Supplement: Supporting Information [file supp_g3.114.012435_FigureS2.pdf]

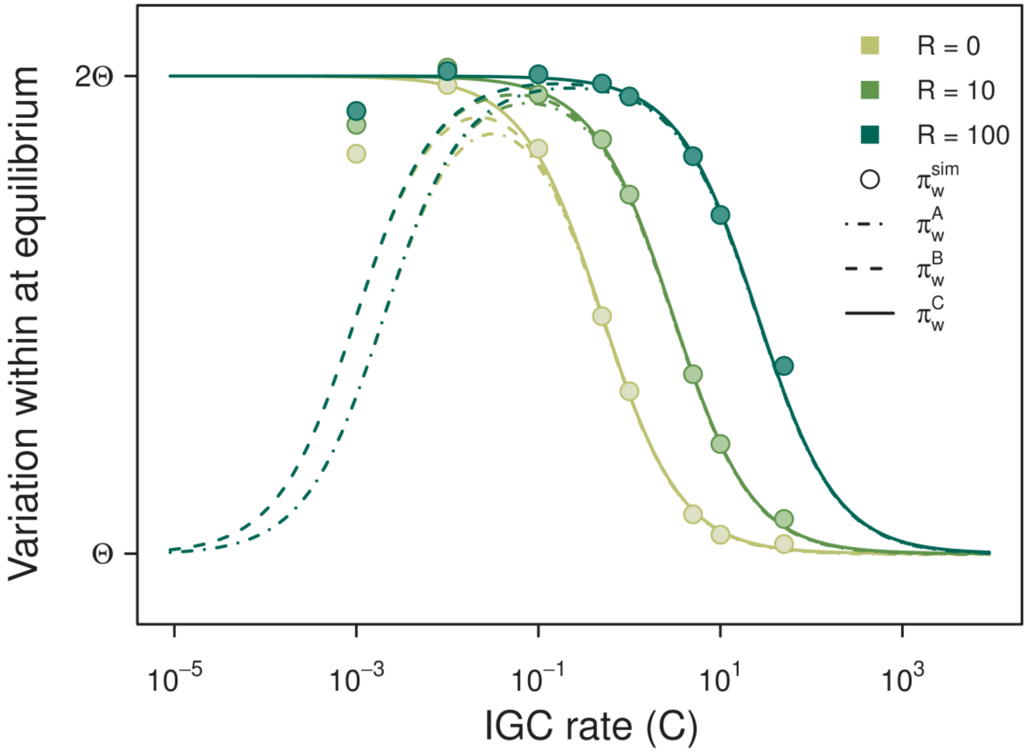

Figure S2 Comparison of variation within blocks between models. Theoretical lines are from Ohta 1982 (π_w^A), Innan 2002 (π_w^B) and Innan 2003 (π_w^C). Circles show results from simulations (π_w^{sim}) with increasingly higher running times in order to reach equilibrium for smaller IGC rates. Simulations were run for 100 thousand generations for *C* = 0.5, 1, 5, 10, 50; 3 million generations for *C* = 0.1, 0.01; and 6 million generations for *C* = 0.001. In the latter case, despite high running times, equilibrium has not yet been reached. Theoretical predictions do not take into account the increasingly high waiting times to reach equilibrium for low IGC rates and thus forward simulations might be very useful to have predictions in this regime.
