# Supplementary material for: Interplay of Interlocus Gene Conversion and Crossover in Segmental Duplications Under a Neutral Scenario
Source: G3 (Bethesda). 2014 Jun 6;4(8):1479–89. doi: 10.1534/g3.114.012435 (PMC4132178; doi:10.1534/g3.114.012435)
Supplement: Supporting Information [file supp_g3.114.012435_FigureS3.pdf]

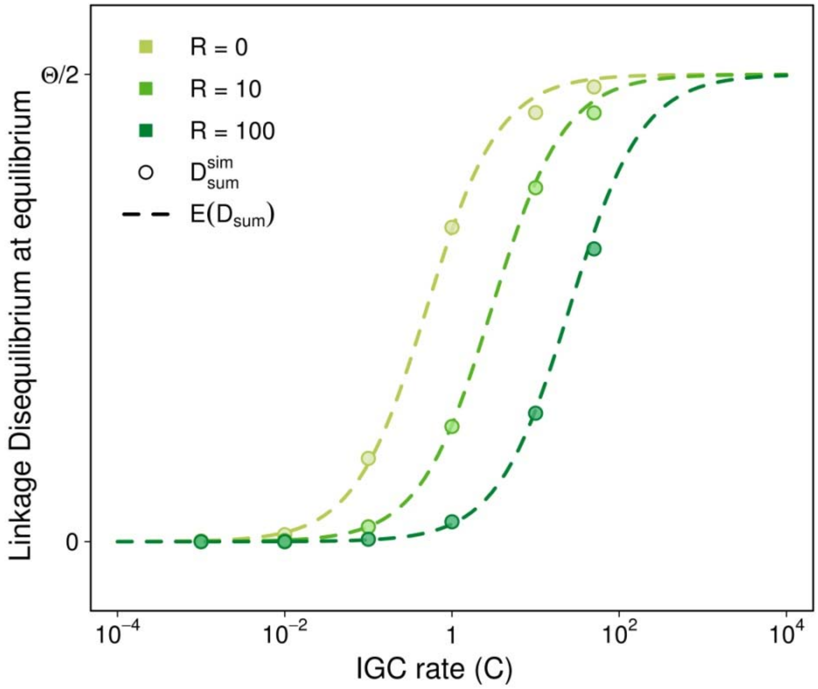

Figure S3 LD between duplicates. The theoretical expectations for LD between duplicates by Innan 2002 ($E(D_{sum})$) are shown together with the corresponding simulation values (D_{sum}^{sim}) for different IGC and crossover rates. LD between segmental duplications increases with IGC and decreases with crossover.
