# Supplementary material for: Interplay of Interlocus Gene Conversion and Crossover in Segmental Duplications Under a Neutral Scenario
Source: G3 (Bethesda). 2014 Jun 6;4(8):1479–89. doi: 10.1534/g3.114.012435 (PMC4132178; doi:10.1534/g3.114.012435)
Supplement: Supporting Information [file supp_g3.114.012435_FigureS4.pdf]

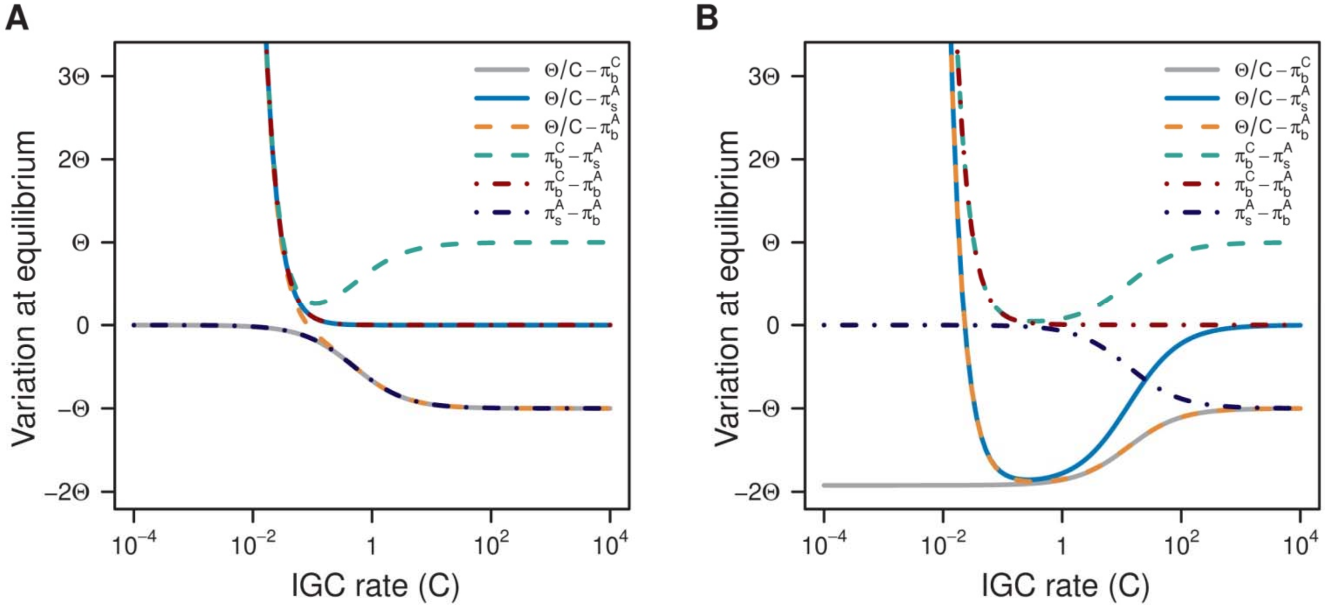

Figure S4 Comparison between different theoretical expectations of variation between duplicates. Figures show expectations for (**A**) R = 0 and (**B**) R = 50. Notice that $\Theta/C - \pi_b^C = \pi_s^A - \pi_b^A$ only for $R = 0$. For $R > 0$, $\Theta/C - \pi_b^C$ decreases with decreasing C as opposed to increasing constantly when $R = 0$. $\pi_s^A - \pi_b^A$, on the other hand, is independent of R.
