# Supplementary material for: Interplay of Interlocus Gene Conversion and Crossover in Segmental Duplications Under a Neutral Scenario
Source: G3 (Bethesda). 2014 Jun 6;4(8):1479–89. doi: 10.1534/g3.114.012435 (PMC4132178; doi:10.1534/g3.114.012435)
Supplement: Supporting Information [file supp_g3.114.012435_FigureS5.pdf]

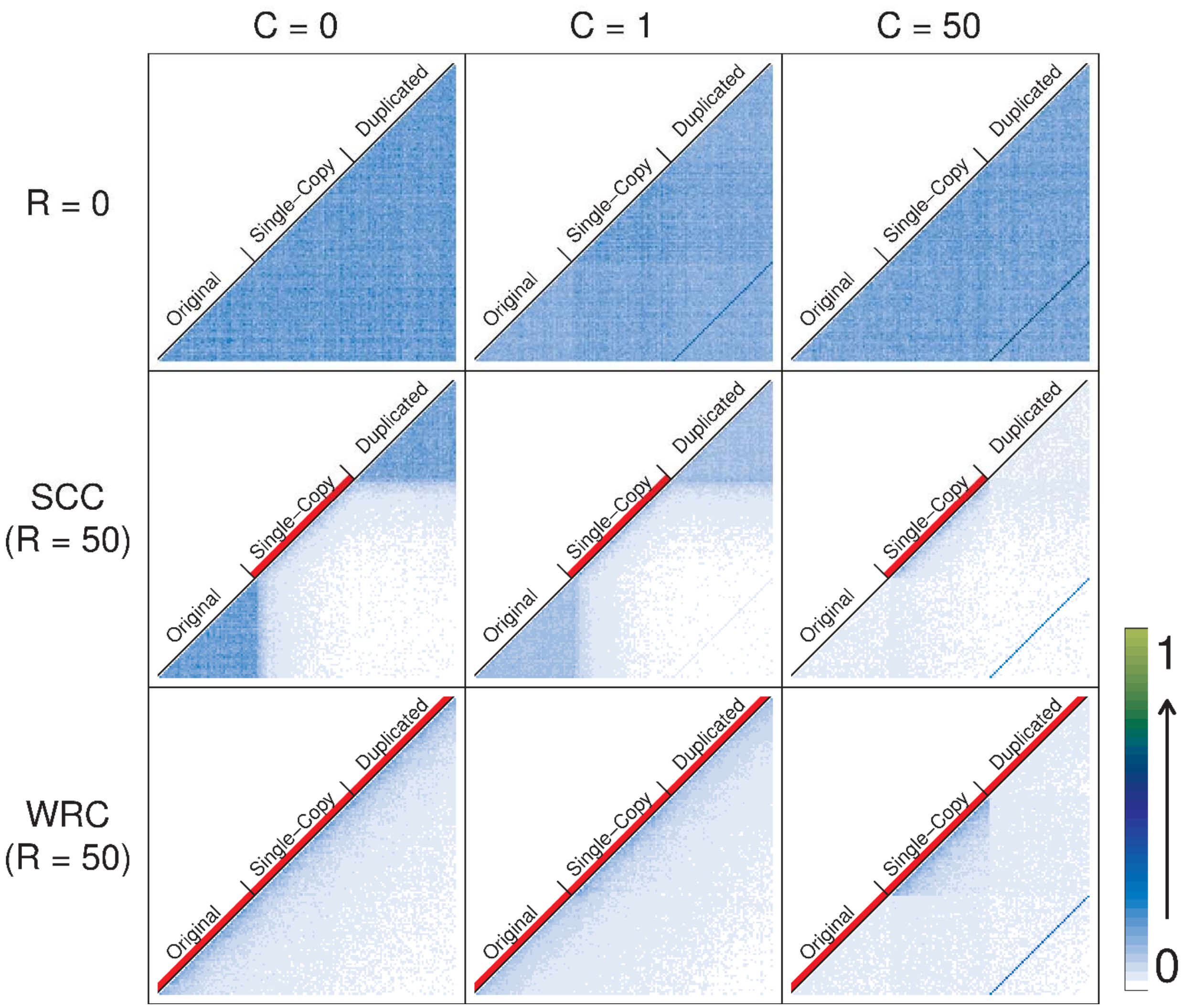

Figure S5 LD along the sequence under different crossover models. Here we use r^2 as a measure of LD. No significant differences can be observed when comparing with D’ in Figure 4.
