# Supplementary material for: Interplay of Interlocus Gene Conversion and Crossover in Segmental Duplications Under a Neutral Scenario
Source: G3 (Bethesda). 2014 Jun 6;4(8):1479–89. doi: 10.1534/g3.114.012435 (PMC4132178; doi:10.1534/g3.114.012435)
Supplement: Supporting Information [file supp_g3.114.012435_FigureS6.pdf]

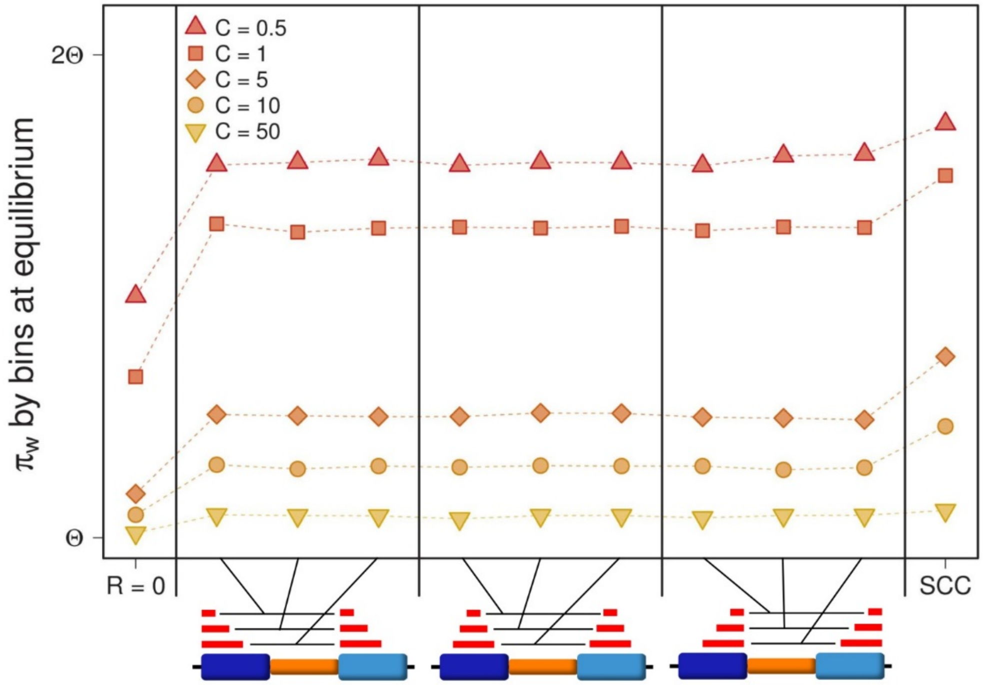

Figure S6 Variation within a duplicate block under different cases of HSC with two hotspots. In contrast to Figure 6 in which cases with a single hotspot are displayed, we show here scenarios with two hotspots present in equivalent locations of the two paralogous copies of the duplication. We observe that when there are two symmetrically located and equally active hotspots, there are no differences in levels of variation along the duplicates. Variation within a duplicate block for all two-hotspot HSC cases fall between the variation within blocks found when no crossover is acting (left) and the variation within blocks found under the SCC model (right), for the same value of *R* (R = 10).
