# Supplementary material for: Interplay of Interlocus Gene Conversion and Crossover in Segmental Duplications Under a Neutral Scenario
Source: G3 (Bethesda). 2014 Jun 6;4(8):1479–89. doi: 10.1534/g3.114.012435 (PMC4132178; doi:10.1534/g3.114.012435)
Supplement: Supporting Information [file supp_g3.114.012435_FigureS7.pdf]

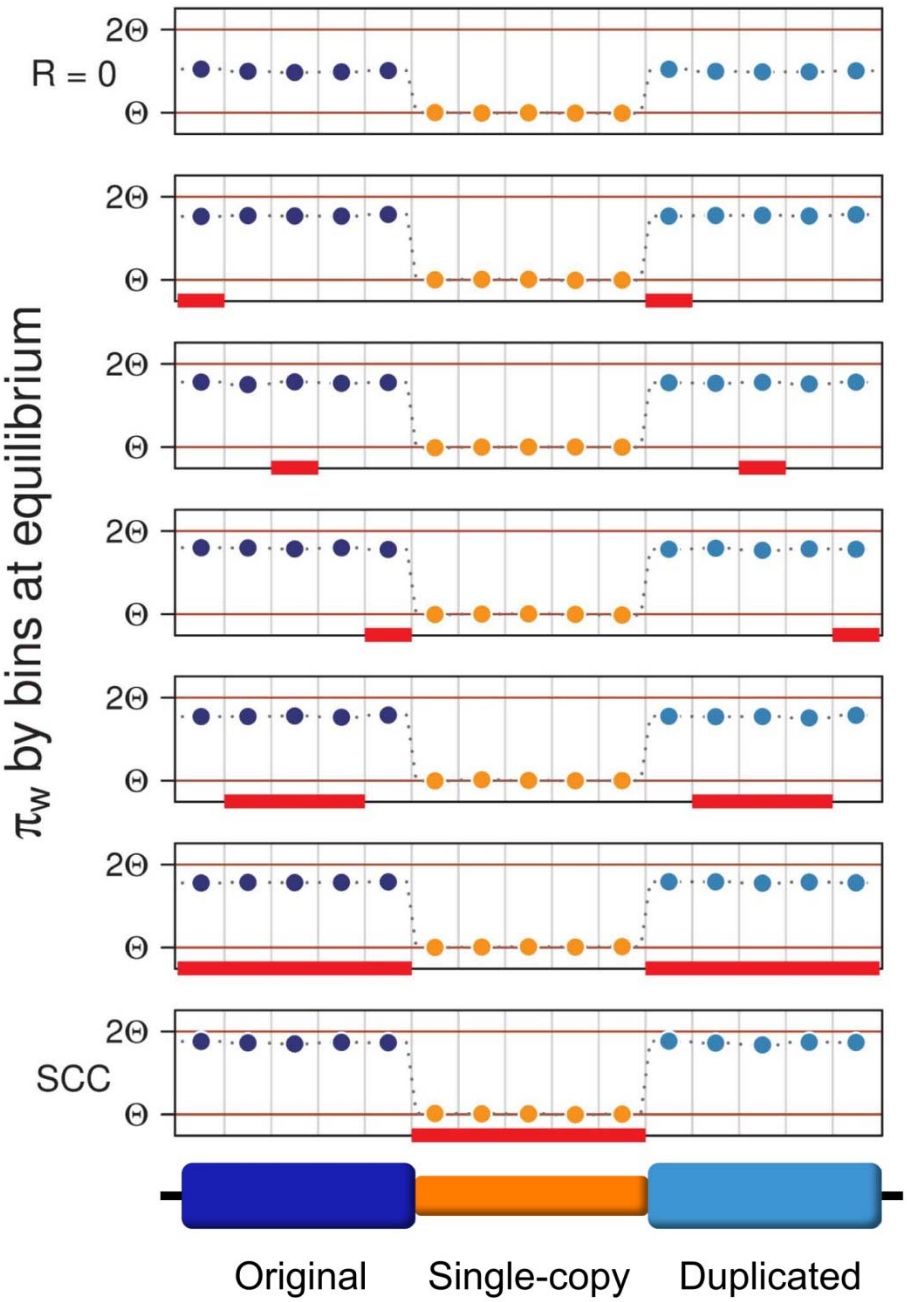

Figure S7 Distribution of variation along the simulated sequence under different two-hotspot HSC cases. The presence of two hotspots in paralogous regions on duplicate blocks homogenizes the amount of variation within all the bins of the duplicated blocks in all cases.
